# Open-Air Chemical Recycling: Fully Oxygen-Tolerant ATRP Depolymerization

**DOI:** 10.1021/jacs.4c05621

**Published:** 2024-07-03

**Authors:** Stella Afroditi Mountaki, Richard Whitfield, Evelina Liarou, Nghia P. Truong, Athina Anastasaki

**Affiliations:** † Laboratory of Polymeric Materials, Department of Materials, ETH Zurich, Zurich 8093, Switzerland; ‡ Department of Chemistry, University of Warwick Library Road, Coventry CV4 7SH, U.K.

## Abstract

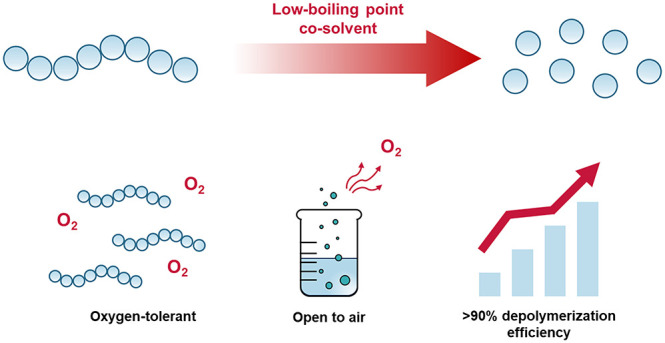

While oxygen-tolerant strategies have been overwhelmingly developed for controlled radical polymerizations, the low radical concentrations typically required for high monomer recovery render oxygen-tolerant solution depolymerizations particularly challenging. Here, an open-air atom transfer radical polymerization (ATRP) depolymerization is presented, whereby a small amount of a volatile cosolvent is introduced as a means to thoroughly remove oxygen. Ultrafast depolymerization (i.e., 2 min) could efficiently proceed in an open vessel, allowing a very high monomer retrieval to be achieved (i.e., ∼91% depolymerization efficiency), on par with that of the fully deoxygenated analogue. Oxygen probe studies combined with detailed depolymerization kinetics revealed the importance of the low-boiling point cosolvent in removing oxygen prior to the reaction, thus facilitating effective open-air depolymerization. The versatility of the methodology was demonstrated by performing reactions with a range of different ligands and at high polymer loadings (1 M monomer repeat unit concentration) without significantly compromising the yield. This approach provides a fully oxygen-tolerant, facile, and efficient route to chemically recycle ATRP-synthesized polymers, enabling exciting new applications.

With the advent of reversible deactivation radical polymerization (RDRP), the synthesis of polymers with controlled dispersity, architecture, sequence, and end-group fidelity has become commonplace.^1−12^ The possibility to pre-install labile end-groups, usually halogens or thiocarbonylthio compounds, not only enables the formation of well-defined block copolymers but also creates the opportunity to trigger low-temperature depolymerization for ATRP- or reversible addition–fragmentation chain transfer (RAFT)-synthesized materials.^13−18^ While earlier reports revealed low monomer conversions during the depolymerization of bulky monomers, subsequent reports sought to intentionally encourage depolymerization under thermodynamically favorable conditions.^19−22^ Ouchi and co-workers first showed that poly(methyl methacrylate) (PMMA) synthesized by ATRP could be depolymerized back to monomers in the presence of ruthenium catalysts, although prevalent side reactions limited the overall monomer regeneration.^23^ The group of Matyjaszewski highlighted the importance of a chlorine end-group to suppress lactonization at high temperatures when either copper or iron catalysts were employed.^24,25^ Our group subsequently showed that bromine-terminated polymers can also be efficiently depolymerized if end-group activation dominates over lactonization.^26^ In the RAFT domain, the Sumerlin group and our group independently demonstrated that solution depolymerization could proceed under either thermal or photothermal conditions by a combination of heat and high dilution.^27−33^ Bulk depolymerization was also feasible by *in situ* removing the monomer during the depolymerization, thereby maximizing the final yield.^34−38^

However, all current depolymerization strategies operate under completely deoxygenated conditions with freeze pump thaw cycles or nitrogen sparging often performed to fully eliminate oxygen prior to depolymerization. This is a crucial step, as oxygen can potentially deactivate the catalyst or react with radicals, leading to terminated polymer chains, which are then unable to depolymerize. Such deoxygenation procedures not only add cost and complexity to these processes but also restrict the widespread applicability of chemical recycling. Instead, oxygen-tolerant strategies have been overwhelmingly developed for controlled radical polymerizations by researchers, including the groups of Matyjaszewski,^39−42^ Boyer,^43−49^ Xu,^45,47,48^ Hawker,^43,50,51^ Haddleton,^52−56^ Chapman,^49,57^ Konkolewicz,^58,59^ and many others.^60−66^ However, oxygen-tolerant depolymerizations are particularly challenging, as the low polymer loadings and the resulting low radical concentrations that are typically required mean that only small amounts of termination can have a significant effect on the overall monomer yield. Therefore, to date, oxygen-tolerant depolymerization remains infeasible. In this work, we develop an open-air depolymerization strategy which yields high monomer recovery and does not require any conventional deoxygenation methods to proceed (Scheme 1). A chlorine-terminated poly(benzyl methacrylate) (PBzMA) was initially synthesized by an optimized activator regenerated by electron transfer (ARGET)-ATRP (Scheme S1) previously developed by Matyjaszewski and co-workers.^24^ The resulting polymer (*Đ* ≈ 1.15, 95% livingness) was subsequently purified and used as a model material for our depolymerization studies (Figures S1–S4). As a control experiment, a fully deoxygenated depolymerization was first attempted under slightly modified literature conditions at 170 °C, using CuCl_2_ and tris(2-pyridylmethyl)amine (TPMA) as the catalyst and 1,2,4-trichlorobenzene (TCB) as the primary solvent. A small amount of a cosolvent was also added (i.e., dimethyl sulfoxide (DMSO) or dimethylformamide (DMF) up to 10% v/v) to aid catalyst dissolution.^20,24^ As expected, when the reaction was degassed with nitrogen and kept sealed under a nitrogen atmosphere, a successful depolymerization could be achieved, reaching 88% (93% efficiency) monomer recovery within 5 min (Figures S5, S7, S8 and Table S1). Instead, when the depolymerization was attempted in open air under otherwise identical conditions, negligible (<1%) monomer regeneration was detected by proton nuclear magnetic resonance (^1^H NMR), even when the reaction was left to proceed for prolonged times (Figures S6–S8 and Table S2). These results demonstrate the expected challenges associated with oxygen-tolerant depolymerizations. To gain a better understanding of the challenges, an optical oxygen sensor was employed to enable online monitoring of the dissolved oxygen within the depolymerization mixture. In the presence of both the catalyst and the cosolvents, the observed oxygen consumption was negligible, indicating that this gas was present throughout the reaction, preventing depolymerization from proceeding (Figure S9).

**Scheme 1:**
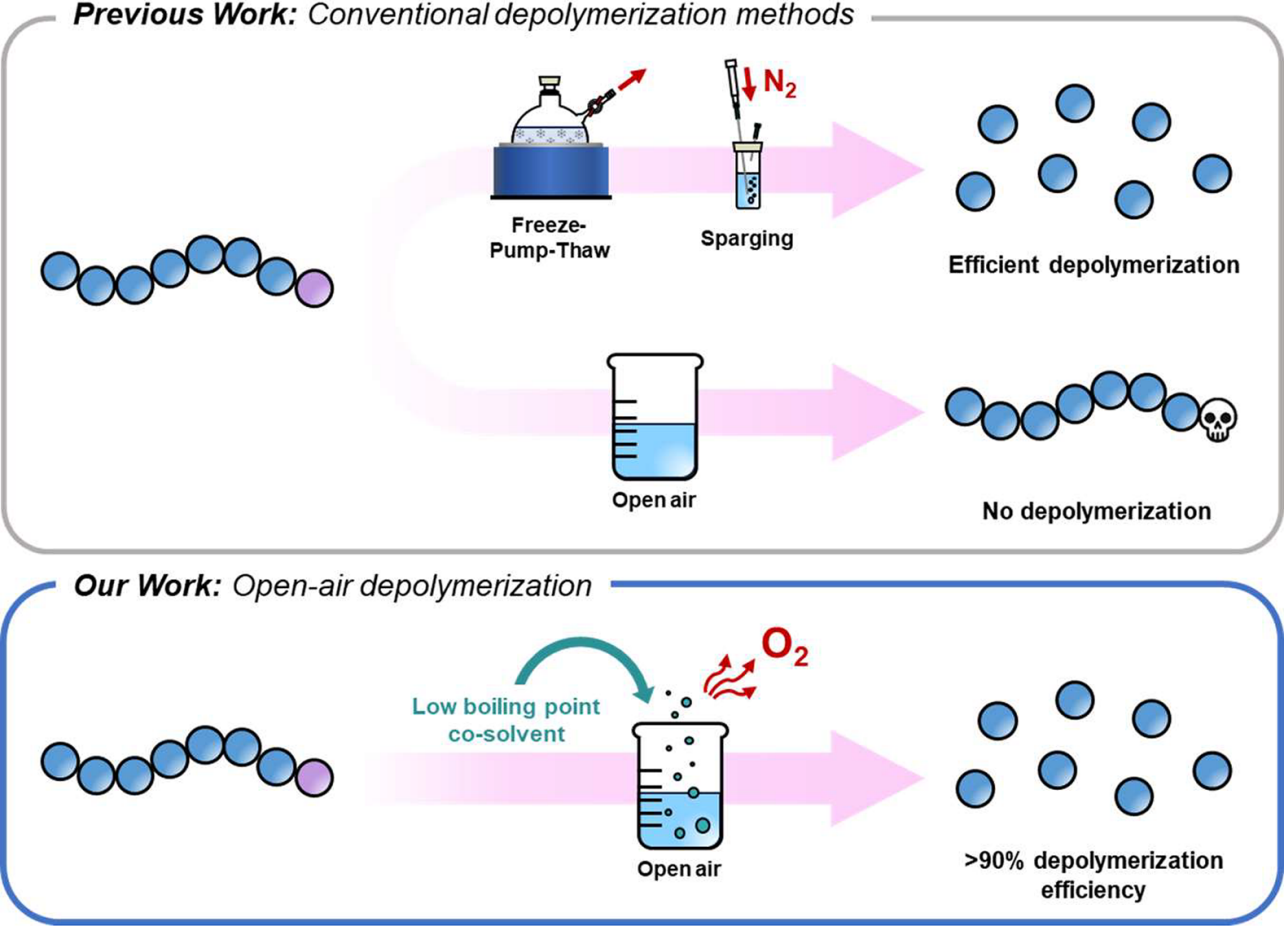
Comparison between Previous Depolymerization Approaches and Our Proposed Open-Air Depolymerization Method

Interestingly, we serendipitously discovered that when the small amount of DMSO or DMF cosolvent was replaced by acetonitrile (MeCN) or acetone, an efficient depolymerization was triggered. In particular, when acetone was employed, 76% of monomer was successfully regenerated after 15 min (80% depolymerization efficiency, Figures 1a,b, S10 and Table S3). Oxygen probe measurements were then conducted, showing that even in the absence of the catalytic system, i.e., in a 10% acetone solution (v/v w.r.t. TCB), the dissolved oxygen could be fully eliminated within 5 min (Figure 1c). A mild boiling of the depolymerization mixture was also visually observed, suggesting that acetone was gradually removed from the system (Figure S11). Inspired by a previous literature report,^67^ we propose that the removal of the oxygen is due to acetone bubbles generated by boiling, which entrain the dissolved oxygen and remove it from the solution. Upon oxygen removal, a rapid depolymerization could then proceed, and oxygen probe measurements revealed that once the acetone had fully evaporated, oxygen could then gradually rediffuse into the reaction (Figure 1c). This oxygen diffusion could be responsible for the cessation of the depolymerization and the slightly lower conversions observed when compared to the fully deoxygenated control experiment (Figure S7). To further investigate our hypothesis, a series of depolymerization reactions was subsequently conducted in the presence of a range of low-boiling point cosolvents (b.p.: 66–118 °C, 10% v/v in TCB). The employed solvents included tetrahydrofuran (THF), isopropanol (IPA), and *n*- butanol (BuOH), and in line with our initial hypothesis, all of these cosolvents facilitated a successful depolymerization, reaching high conversions (i.e., 71–80% depolymerization efficiency, Figures 1d, S12 and Table S4). At the same time, when further high-boiling point cosolvents were investigated, i.e., xylene, chlorobenzene (PhCl), and tetraethylene glycol dimethyl ether (TEGDME) (PhCl, b.p. of 132 °C; xylene, b.p. of 139 °C; and TEGDME, b.p. of 275 °C), minimal, if any, depolymerization could be detected by ^1^H NMR (Figures 1e, S12 and Table S4). Taken altogether, these results clearly suggest that an inexpensive low-boiling point cosolvent, such as acetone, can rapidly remove oxygen via a mild boiling enabling an efficient depolymerization reaction to proceed. Since the highest conversion reached thus far was 76% (80% depolymerization efficiency), which was slightly lower than when the depolymerization proceeded in an inert atmosphere (i.e., 89%), we sought to further optimize our system by exploring the effect of the amount of cosolvent. Specifically, it was postulated that the higher the amount of cosolvent, the faster the oxygen removal would take place, thus leading to more efficient depolymerizations. To explore this, the depolymerization was first studied using 50 μL of acetone (5% v/v w.r.t. TCB), as this was the lowest possible amount required to dissolve the catalyst (Figure 2a,b and Table S5). Relatively low monomer recovery was observed (∼30%, 31% depolymerization efficiency), and an oxygen probe measurement of this solvent mixture showed that a total of 5 min was needed for the oxygen content to decrease to negligible levels (Figure 2c). Instead, by gradually increasing the cosolvent content, higher overall depolymerization yields were achieved. For instance, 200 μL of acetone (20% v/v) gave rise to 81% monomer regeneration, and this value further increased to 86% (85–91% depolymerization efficiency, respectively) when 300 μL of acetone was employed (Table S5). We attribute the nonquantitative depolymerization (∼91%) to a small amount of lactonization, as evidenced in Figure S13. To further rationalize these results, additional oxygen probe measurements were performed. Figure 2c shows that by increasing the cosolvent concentration, faster oxygen removal was evident, while Figure S14 confirms that the key parameter for the oxygen removal was the evaporation of the volatile cosolvent, not the presence of the catalyst. Among all cosolvents, only the highest acetone loading (i.e., 30% v/v) fully eliminated oxygen, and this was also the experiment where the highest depolymerization conversion was achieved. A detailed depolymerization kinetic analysis was subsequently performed for this reaction (Figures 2d, S15 and Table S6). In the first 2 min, when oxygen was still present in the reaction mixture, no meaningful depolymerization took place (i.e., <5% monomer regeneration). However, upon successful removal of the dissolved oxygen, a rapid depolymerization was initiated, which required only 2 min to be completed. At the same time, after 3 min, we observed that oxygen was diffusing back into the reaction mixture. These results highlight that an open-air depolymerization is only feasible due to the rapid oxygen removal enabled by the addition of a cosolvent combined with the ultrafast nature of the depolymerization that reaches near-quantitative conversion prior to oxygen rediffusion.

**Figure 1:**
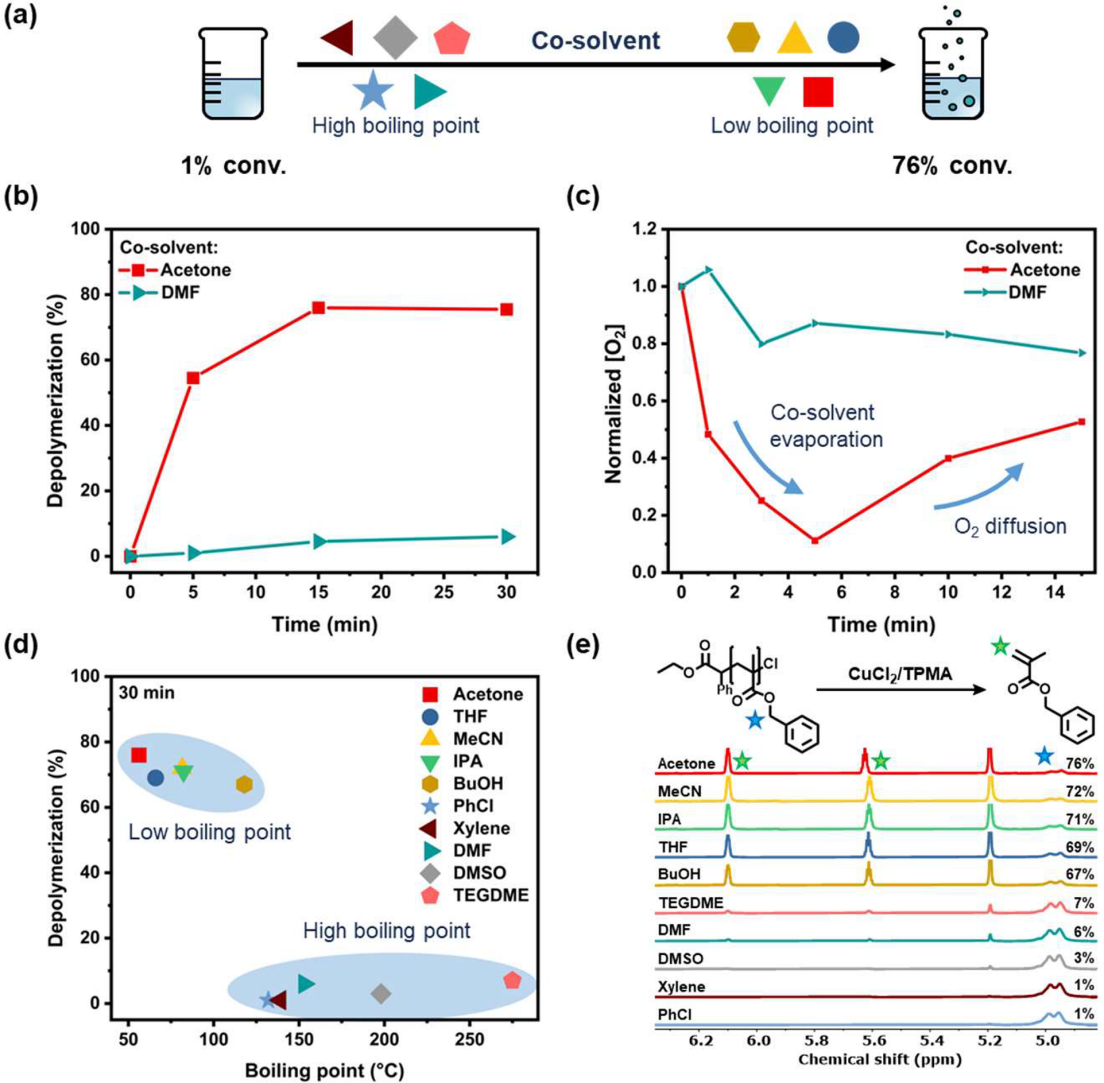
(a) Schematic representation of the effect of various cosolvents on the open-air depolymerization conversion. (b) Depolymerization kinetics comparing reactions performed with acetone and DMF as cosolvents. (c) O_2_ concentration measurements comparing acetone and DMF as cosolvents. (d) Scatter plot illustrating the effect of cosolvent boiling point on the final depolymerization conversion. (e) ^1^H NMR spectra of reactions containing various cosolvents after 30 min of reaction time. All reactions were carried out at 170 °C with a 50 mM repeat unit concentration of polymer and 10% v/v cosolvent content in TCB.

**Figure 2:**
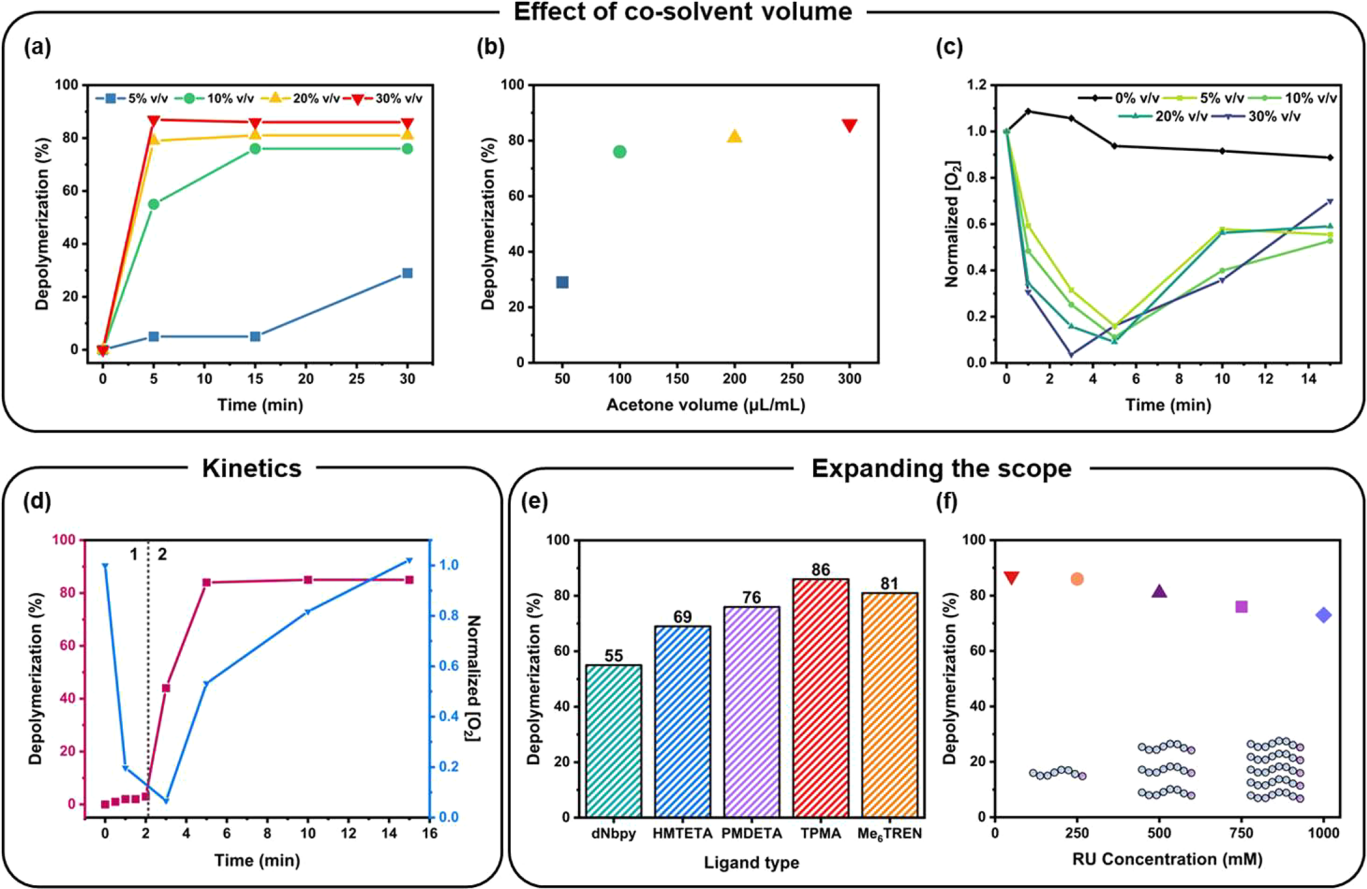
(a) Depolymerization kinetics, (b) maximum conversion after 30 min of depolymerization, and (c) O_2_ concentration measurement for experiments performed with various acetone contents. (d) The evolution of depolymerization conversion and O_2_ concentration with time under the optimized cosolvent content (30% v/v acetone). Effect of (e) different ATRP ligands and (f) polymer loadings. Reactions were run at 170 °C with 70% v/v TCB.

To expand the scope of the depolymerization reaction, first a higher molecular weight poly(benzyl methacrylate) was synthesized by ARGET-ATRP (*M*_n_ = 18,000). Depolymerization was performed, and upon increasing the catalyst concentration (P-Cl:CuCl_2_:TPMA = 1:1.2:7), as much as 76% of the starting monomer could be regenerated (Figures S16, S17 and Table S7). Next, alternate polymers were investigated. Poly(methyl methacrylate) and poly(butyl methacrylate) were prepared and subsequently utilized for open-air depolymerization under our previously optimized conditions (P-Cl:CuCl_2_:TPMA = 1:0.22:1.3, Figures S18–S21). Pleasingly, in both cases, we could obtain very high depolymerization conversions in just 15 min (89% and 88%, respectively, Figures S22–S24 and Table S8). Subsequently, the compatibility of the depolymerization with various nitrogen-containing ligands was explored (Figures 2e, S25 and Table S9). Ligands capable of forming high activity complexes, such as TPMA and tris 2-(dimethylamino)ethyl amine (Me_6_Tren) generated the highest monomer recovery (81–86%, 85–91% depolymerization efficiency), while ligands that formed lower activity complexes, including 1,1,4,7,10,10-hexamethyltriethylenetetramine (HMTETA) and *N*,*N*,*N*′,*N*″,*N*″-pentamethyl diethylenetriamine (PMDETA), also enabled efficient depolymerization, albeit with slightly lower yields. In particular, when the inexpensive and commercially available ligand PMDETA was employed, 76% (80% depolymerization efficiency) of monomer could be generated. Instead, when the lowest activity complex, containing 4,4′-dinonyl-2,2′-dipyridyl (dNbpy), was used, only 55% conversion (58% depolymerization efficiency) was recorded, suggesting that higher activity complexes are better suited for open-air depolymerizations. We attribute this to the fast activation needed to trigger a rapid depolymerization, which must be complete prior to the oxygen rediffusion. Finally, we examined the possibility of our open-air depolymerization operating at higher polymer loadings (Figures 2f, S26 and Table S10). Pleasingly, while a 50 mM initial monomer repeat unit concentration led to 87% (92% depolymerization efficiency) depolymerization, very little decrease in the final yield was observed at higher initial concentrations. For instance, at 250 mM, a comparable 86% (91% depolymerization efficiency) conversion was recorded, while at 500 mM, 81% (85% depolymerization efficiency) monomer recovery was obtained. Notably, at even higher initial repeat unit concentrations (i.e., 750 and 1 M), 76% and 73% depolymerization yields were achieved, respectively (80 and 77% depolymerization efficiency, respectively). These findings highlight the superiority of open-air ATRP depolymerization to operate at higher concentrations, in contrast to previous RAFT depolymerization protocols, which necessitate low initial monomer concentrations (i.e., 5 mM) to proceed.^22^

To summarize, in this work, we present the first example of an open-air ATRP depolymerization that operates under a range of reaction conditions and polymer loadings. The key to our approach is to use a small amount of an inexpensive, low-boiling point cosolvent, such as acetone, to *in situ* remove the oxygen, allowing depolymerization to commence. These findings were further rationalized by utilizing an oxygen probe in combination with detailed depolymerization kinetics. Under judiciously optimized conditions, very high depolymerization yields can be achieved in an open vessel, thus circumventing the need to thoroughly deoxygenate the depolymerization mixtures. We envision that this approach will provide an ultrafast, facile, and oxygen-tolerant methodology to both experts and nonexperts.

## Supporting Information Available

General information, experimental procedures, and various spectroscopic data (PDF)
