# Supplementary material for: Open-Air Chemical Recycling: Fully Oxygen-Tolerant ATRP Depolymerization
Source: J Am Chem Soc. 2024 Jul 3;146(28):18848–54. doi: 10.1021/jacs.4c05621 (PMC11258787; doi:10.1021/jacs.4c05621)
Supplement: Supplementary file 1 — ja4c05621_si_001.pdf [file ja4c05621_si_001.pdf]

Open-Air Chemical Recycling: Fully Oxygen Tolerant ATRP Depolymerization

Stella Afroditi Mountaki [a], Richard Whitfield [a], Evelina Liarou [b], Nghia P. Truong [a], Athina Anastasaki *[a]

[a] Laboratory of Polymeric Materials, Department of Materials, ETH Zurich, Zurich 8093, Switzerland

[b] Department of Chemistry, University of Warwick Library Road, Coventry, CV4 7AL, UK

Contents

Materials 3
Proton nuclear magnetic resonance (^{1}H NMR) 3
Size exclusion chromatography (SEC) 3
Oxygen probe 3
General procedures 4
Synthesis of PBzMA-Cl by ARGET-ATRP 4
Chain extension of PBzMA-Cl by ARGET-ATRP 5
General procedure for deoxygenated depolymerization of PBzMA-Cl using DMF as the co-solvent 6
General procedure for open-air depolymerization of PBzMA-Cl using acetone as the co-solvent 6
General procedure for open-air depolymerization of PMMA-Cl 6
General procedure for open-air depolymerization of PBMA-Cl 7
Kinetic analysis 7
Oxygen probe measurements to test co-solvents (Figure 1c) 7
Oxygen probe measurements for optimized conditions (Figure 2d) 7
Additional characterization data 9
Polymer synthesis of PBzMA-Cl by ARGET-ATRP 9
Livingness quantification 11
Deoxygenated depolymerization of PBzMA-Cl 13
Open-air depolymerization of PBzMA-Cl under previously optimized conditions 15
Open-air depolymerization in the presence of low and high boiling-point co-solvents 19
Depolymerization kinetics of PBzMA-Cl 26
Expanding the polymer scope of open-air depolymerization 28
Polymer synthesis of higher molecular weight PBzMA-Cl by ARGET-ATRP 28
Depolymerization of higher molecular weight PBzMA-Cl 29
Polymer synthesis of PMMA-Cl by ARGET-ATRP 31
Polymer synthesis of PBMA-Cl by ARGET-ATRP 33
Depolymerization of PMMA-Cl and PBMA-Cl 35
Expanding the ligand compatibility of open-air depolymerization 39
The effect of the repeat unit concentration on the open-air depolymerization 41

Materials

Benzyl methacrylate (>98.0%) and tris(2-pyridylmethyl)amine were obtained from Tokyo Chemical Industry. Ethyl α-chlorophenyl acetate (97%) and 1,2,4-trichlorobenzene were obtained from Acros Organics. *N*,*N*-Dimethylformamide (>99%) was obtained from Fluka. The remaining chemicals were purchased from Sigma-Aldrich. Tris-(2-(dimethylamino)ethyl)amine was synthesized according to the literature and stored in the fridge.[1] All chemicals were used as received, except the monomer that was passed through a column of basic alumina prior to usage.

Proton nuclear magnetic resonance (^{1}H NMR)

^{1}H NMR spectra were measured on a Bruker DPX – 300 spectrometer with deuterated acetone (acetone-d6) as the solvent. The depolymerization conversions were assessed by comparing the integrals of the monomeric vinyl protons (5.64–6.22 ppm) to the integrals of both the monomer and polymer peaks (4.94–5.26 ppm).

Size exclusion chromatography (SEC)

SEC was performed using a Shimadzu modular system consisting of a CBM-20A system controller, an SIL-20A automatic injector, a 10.0 μm bead size guard column (50 × 7.5 mm), followed by three KF-805L columns (300 × 8 mm, bead size: 10 μm, pore size maximum: 5000 Å) and an RID-20A differential refractive-index detector. *N*,*N*-Dimethylacetamide (HPLC grade, with 0.03% w/v LiBr) served as the eluent and the flow rate was maintained at 1 mL min^{-1} using an LC-20AD pump. A calibration curve for molecular weight was generated using commercially available narrow molar mass distribution poly(methyl methacrylate) standards with molecular weights spanning from 5000 to 1.5 × 10^6 Da. SEC analysis was carried out in dimethylacetamide, after samples had been passed through a column of basic alumina and a 0.45 μm PTFE filter.

Oxygen probe

A Pocket Oxygen Meter - FireStingGO2 (from Pyro Science) was used for all measurements. The solvent-resistant oxygen probe OXSOLV measures oxygen partial pressure in various polar and nonpolar solvents, based on optical detection principles (REDFLASH technology). The fiber-optic oxygen sensor tip is covered with a stainless-steel tube 1.5 mm in diameter and 150 (or 40) mm in length. The analysis of the data was conducted with the FireStingGO2 Manager software.

General procedures

Synthesis of PBzMA-Cl by ARGET-ATRP

In a round bottom flask, equipped with a stirrer bar, 79.3 mg (0.59 mmol, 0.2 equiv.) of $CuCl_2$ was dissolved in acetonitrile (40 mL) alongside 123 µL (0.59 mmol, 0.2 equiv.) of N,N,N′,N″,N″-pentamethyldiethylenetriainine (PMDETA). The solution was then sonicated for 1 min to ensure full solubilization of the copper salt, resulting in a light blue solution. Next, 507 µL (2.95 mmol, 1 equiv.) of ethyl α-chlorophenyl acetate (ECPA) and 50 mL (0.295 mol, 100 equiv.) of benzyl methacrylate (BzMA) were added to the flask, which was sealed with a rubber septum and deoxygenated via nitrogen purging for 20 minutes. After deoxygenation, 76.5 µL (0.24 mmol, 0.08 equiv.) of pre-deoxygenated tin(II) 2-ethylhexanoate ($Sn(EH)_2$) was added, and the color of the solution immediately intensified resulting in indigo blue color solution. The reaction flask was immersed in a pre-heated oil bath at 70 °C and constantly stirred for 2 hours. The reaction was subsequently stopped by opening it to air and samples were taken to measure ^{1}H NMR and SEC. The rest of the polymerization solution was diluted in acetone and passed through a basic alumina column to remove the catalyst. This solution was then concentrated and precipitated into cold methanol. The isolated polymer was dried in a vacuum oven at room temperature for 48 hours and then analyzed again with ^{1}H NMR and SEC to ensure its purity and that the desired final molecular weight and dispersity had been obtained. For the synthesis of the high molecular weight PBzMA-Cl, a similar procedure was followed and the ratio of the components was adjusted to [BzMA]:[ECPA]:[$CuCl_2$]:[PMDETA]:[$Sn(EH)_2$] = 240:1:0.1:0.15:0.2.

Synthesis of PMMA-Cl by ARGET-ATRP

In a 15 mL vial, equipped with a stirrer bar, 5 mg (3.8×10^{-5} mol, 0.1 equiv.) of $CuCl_2$ was dissolved in a 0.5:9.5 acetonitrile:anisole solution (1.5 µL total) alongside 12 µL (5.6×10^{-5} mol, 0.15 equiv.) of N,N,N′,N″,N″-pentamethyldiethylenetriainine (PMDETA). Next, 64 µL (3.8×10^{-4} mol, 1 equiv.) of ethyl α-chlorophenyl acetate (ECPA) and 4 mL (3.8×10^{-2} mol, 100 equiv.) of methyl methacrylate (MMA) were added to the flask, which was sealed with a rubber septum and deoxygenated via nitrogen purging for 20 minutes. In another vial, 24 µL (7.5×10^{-5} mol, 0.2 equiv.) of $Sn(EH)_2$ was dissolved in 500 µL of anisole and subsequently degassed via nitrogen purging for 20 min. Lastly, the 500 µL of the $Sn(EH)_2$ solution were transferred to the first vial and the color of the solution changed to clear blue. The reaction flask was immersed in a pre-heated oil bath at 70 °C and constantly stirred for 2 hours. The reaction was subsequently stopped by opening it to air and samples were taken to measure ^{1}H NMR and SEC. The rest of the polymerization solution was diluted in acetone and passed through a basic alumina column to remove the catalyst. This solution was then concentrated and precipitated into cold methanol. The isolated polymer was dried in a vacuum oven at room temperature for 48 hours and then analyzed

again with ^{1}H NMR and SEC to ensure its purity and that the desired final molecular weight and dispersity had been obtained.

Synthesis of PBMA-Cl by ARGET-ATRP
In a 15 mL vial, equipped with a stirrer bar, 3.4 mg (2.5 x 10^{-5} mol, 0.1 equiv.) of $CuCl_2$ was dissolved in a 0.5:9.5 acetonitrile:anisole solution (1.5 μL total) alongside 8 μL (3.8 x 10^{-5} mol, 0.15 equiv.) of N,N,N′,N″,N″-pentamethyldiethylenetriainine (PMDETA). Next, 43 μL (2.5 x 10^{-4} mol, 1 equiv.) of ethyl α-chlorophenyl acetate (ECPA) and 4 mL (2.5 x 10^{-2} mol, 100 equiv.) of methyl methacrylate (MMA) were added to the flask, which was sealed with a rubber septum and deoxygenated via nitrogen purging for 20 minutes. In another vial, 16 μL (5 x 10^{-5} mol, 0.2 equiv.) of $Sn(EH)_2$ was dissolved in 500 μL of anisole and subsequently degassed via nitrogen purging for 20 min. Lastly, the 500 μL of the $Sn(EH)_2$ solution were transferred to the first vial and the color of the solution changed to clear blue. The reaction flask was immersed in a pre-heated oil bath at 70 °C and constantly stirred for 2 hours. The reaction was subsequently stopped by opening it to air and samples were taken to measure ^{1}H NMR and SEC. The rest of the polymerization solution was diluted in acetone and passed through a basic alumina column to remove the catalyst. This solution was then concentrated and precipitated into cold methanol. The isolated polymer was dried in a vacuum oven at room temperature for 48 hours and then analyzed again with ^{1}H NMR and SEC to ensure its purity and that the desired final molecular weight and dispersity had been obtained.

Chain extension of PBzMA-Cl by ARGET-ATRP
In a 6 mL glass vial, equipped with a stirrer bar, 0.10 mg (7.5 x 10^{-7} mol, 0.05 equiv.) of $CuCl_2$ and 0.47 μL (2.2 x 10^{-6} mol, 0.15 equiv.) of PMDETA were dissolved in a 0.5:9.5 acetonitrile:anisole solution (200 μL total). Next, 100.1 mg (1.5 x 10^{-5} mol, 1 equiv.) of the PBzMA-Cl were weighed and placed in the vial, alongside 510 μL (3 x 10^{-3} mol, 200 equiv.) of BzMA. The content of the vial was stirred until total dissolution had occurred. In another vial, 0.97 μL (3 x 10^{-6} mol, 0.2 equiv.) of $Sn(EH)_2$ was dissolved in 50 μL of anisole. Both vials were degassed via nitrogen purging for 20 min. Lastly, the 50 μL of the $Sn(EH)_2$ solution were transferred to the first vial and the color of the solution changed to clear blue. The reaction was then placed in a preheated oil bath at 70 °C and left to react under constant stirring for 2.5 hours. To stop the reaction, the vial was opened to air prior to ^{1}H NMR and SEC analysis.

Livingness Calculation
% Livingness = the number of living polymer chains/the total number of polymer chains x100%.

This calculation is based on the efficiency of chain extension of the polymer. Please see figures S3 and S4 for further information.

Depolymerization Efficiency Calculation

% Depolymerization Efficiency = the amount of monomer obtained during the depolymerization/maximum possible amount of monomer obtainable on full depolymerization of all living polymer chains x 100%.

General procedure for deoxygenated depolymerization of PBzMA-Cl using DMF as the co-solvent

In a 15 mL vial, 2 mg (1.5 x 10^{-5} mol) of $CuCl_2$ and 25.5 mg (9 x 10^{-5} mol) of tris(2-pyridylmethyl) amine (TPMA) were placed. To dissolve the copper complex, 5 mL of DMF were added. In a 15 mL glass test tube, 9.1 mg (1.4 x 10^{-6} mol, 1 equiv.) of PBzMA-Cl and 900 µL 1,2,4-trichlorobenzene (TCB) were introduced. 100 µL (0.04 mg $CuCl_2$, 3 x 10^{-7} mol, 0.22 equiv., 0.51 mg TPMA, 1.8 x 10^{-6} mol, 1.3 equiv.) of the DMF catalyst stock solution was introduced to the reaction tube and it was stirred vigorously to dissolve all solids. The test tube was sealed with a rubber septum and then bubbled with nitrogen for 30 minutes. A small aliquot of the reaction was collected, for ^{1}H NMR and SEC analysis and the reaction tube was placed into the oil bath at 170 °C. Additional samples were collected after 5, 15, and 30 minutes of reaction time. For different co-solvent contents, the ratio of solvent to co-solvent introduced into the reaction was adjusted, while keeping the concentration of all other components consistent.

General procedure for open-air depolymerization of PBzMA-Cl using acetone as the co-solvent

In a 15 mL vial, 2 mg (1.5 x 10^{-5} mol) of $CuCl_2$ and 25.5 mg (9 x 10^{-5} mol) of tris(2-pyridylmethyl) amine (TPMA) were placed. To dissolve the copper complex, 5 mL of acetone were added. In a separate 15 mL glass test tube, 9.1 mg (1.4 x 10^{-6} mol, 1 equiv.) of PBzMA-Cl and 900 µL 1,2,4-trichlorobenzene (TCB) were introduced. 100 µL (0.04 mg $CuCl_2$, 3 x 10^{-7} mol, 0.22 equiv., 0.51 mg TPMA, 1.8 x 10^{-6} mol, 1.3 equiv.) of the acetone catalyst stock solution was introduced to the reaction tube and it was stirred vigorously to dissolve all solids. A small aliquot of the reaction was collected, for ^{1}H NMR and SEC analysis and the reaction tube was placed into the oil bath at 170 °C. Additional samples were collected after 5, 15, and 30 minutes of reaction time. For different co-solvent contents, the ratio of solvent to co-solvent introduced into the reaction was adjusted, while keeping the concentration of all other components consistent.

General procedure for open-air depolymerization of PMMA-Cl

In a 30 mL vial, 3.2 mg (2.4 x 10^{-5} mol) of $CuCl_2$ and 41 mg (1.4 x 10^{-4} mol) of tris(2-pyridylmethyl) amine (TPMA) were placed. To dissolve the copper complex, 30 mL of acetone were added. In a separate 15 mL glass test tube, 5.2 mg (1.1 x 10^{-6} mol, 1 equiv.) of PMMA-Cl and 700 µL 1,2,4-trichlorobenzene (TCB) were introduced. 300 µL (0.03 mg $CuCl_2$, 2.4 x 10^{-7} mol, 0.22 equiv., 0.41 mg TPMA, 1.4 x 10^{-6} mol, 1.3 equiv.) of the acetone catalyst stock solution was introduced to the reaction tube and it was stirred vigorously to dissolve all solids. Furthermore, 15 µL of $PEG_{350,}$ were added to the reaction

solution, so as to serve as an internal standard. A small aliquot of the reaction was collected, for ^{1}H NMR analysis and the reaction tube was placed into the oil bath at 170 °C. Additional samples were collected after 5, 15, and 30 minutes of reaction time. All samples were dried under air flow prior to analysis.

General procedure for open-air depolymerization of PBMA-Cl

In a 30 mL vial, 2.8 mg (2.1 x 10^{-5} mol) of $CuCl_2$ and 36 mg (1.2 x 10^{-4} mol) of tris(2-pyridylmethyl) amine (TPMA) were placed. To dissolve the copper complex, 30 mL of acetone were added. In a separate 15 mL glass test tube, 7.3 mg (9.4 x 10^{-7} mol, 1 equiv.) of PBMA-Cl and 700 µL 1,2,4-trichlorobenzene (TCB) were introduced. 300 µL (0.03 mg $CuCl_2$, 2.1 x 10^{-7} mol, 0.22 equiv., 0.36 mg TPMA, 1.2 x 10^{-6} mol, 1.3 equiv.) of the acetone catalyst stock solution was introduced to the reaction tube and it was stirred vigorously to dissolve all solids. Furthermore, 15 µL of $PEG_{350,}$ were added to the reaction solution so as to serve as an internal standard. A small aliquot of the reaction was collected, for ^{1}H NMR analysis and the reaction tube was placed into the oil bath at 170 °C. Additional samples were collected after 5, 15, and 30 minutes of reaction time. All samples were dried under air flow prior to analysis.

Kinetic analysis

In a 30 mL glass test tube, 2 mg (1.5 x 10^{-5} mol) of $CuCl_2$ and 25.5 mg (9 x 10^{-5} mol) of tris(2-pyridylmethyl) amine (TPMA) were placed. To dissolve the copper complex, 15 mL of acetone were added. In a separate 15 mL glass tube, 9.1 mg (1.4 x 10^{-6} mol, 1 equiv.) of PBzMA-Cl and 700 µL 1,2,4-trichlorobenzene (TCB) were introduced. 300 µL (0.04 mg $CuCl_2$, 3 x 10^{-7} mol, 0.22 equiv., 0.51 mg TPMA, 1.8 x 10^{-6} mol, 1.3 equiv.) of the acetone catalyst stock solution was introduced to the reaction tube and it was stirred vigorously to dissolve all solids. A small aliquot of the reaction was collected, for ^{1}H NMR and SEC analysis and the reaction tube was placed into the oil bath at 170 °C. Additional samples were collected after 0.5, 1, 1.5, 2, 3, 5, 10 and 15 minutes of reaction time for ^{1}H NMR, SEC analysis and O_2 content measurements.

Oxygen probe measurements to test co-solvents (Figure 1c)

Into a 15 mL vial, 4.5 mL of TCB and 0.5 mL of co-solvent were added. This solution was then divided equally between five separate test tubes (1 mL per tube). The oxygen sensor was then immersed in one of the test tubes and a measurement was taken (t=0). The 5 unsealed test tubes were then placed into an oil bath at 170 °C for 1, 3, 5, 10 and 15 minutes. After the prescribed amount of time, each test tube was removed in turn from the oil bath, instantly sealed, and cooled in an ice bath for 15 seconds and then an oxygen probe measurement was taken.

Oxygen probe measurements for optimized conditions (Figure 2d)

Into a 15 mL vial, 45.5 mg of PBzMA-Cl (6.8 x 10^{-6} mol, 1 equiv.) was dissolved in 3.5 mL of TCB. In parallel, in a second vial, 0.20 mg (1.5 x 10^{-6} mol, 0.22 equiv.) of $CuCl_2$ and 2.55 mg (9.0 x 10^{-6} mol, 1.3 equiv.) of TPMA were solubilized in 1.5 mL of acetone. These

two solutions were then added together and the mixture was then divided equally between five separate test tubes (1 mL per tube). The oxygen sensor was then immersed in one of the test tubes and a measurement was taken (t=0). The 5 test tubes were then placed into an oil bath at 170 °C for 1, 3, 5, 10 and 15 minutes. After the prescribed amount of time, each test tube was removed in turn from the oil bath, instantly sealed, and cooled in an ice bath for 15 seconds and then an oxygen probe measurement was taken.

Additional characterization data

Polymer synthesis of PBzMA-Cl by ARGET-ATRP

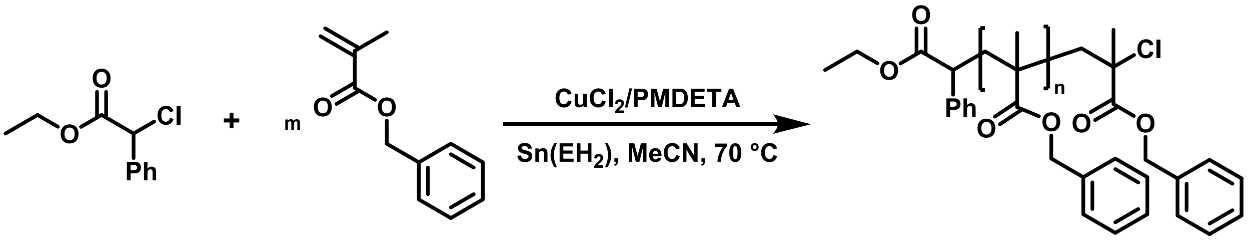

Scheme S1: Schematic representation of polymerization of BzMA via ARGET-ATRP under the following conditions: [ECPA]:[BzMA]:[$CuCl_2$]:[PMDETA]:[$Sn(EH)_2$] = 1:100:0.2:0.2:0.08 in acetonitrile (1:1.25 solvent to monomer ratio) at 70 °C.

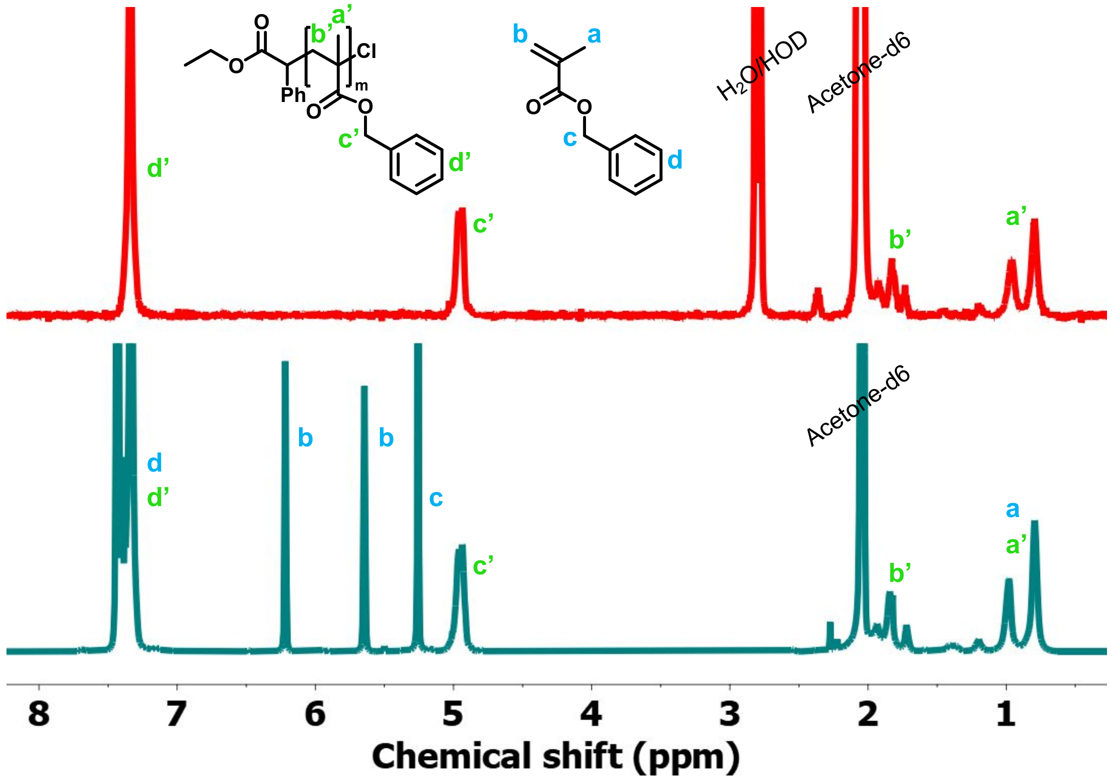

Figure S1: ^{1}H NMR spectra of PBzMA-Cl before (bottom) and after (top) purification. It was synthesized by ARGET-ATRP under the following conditions: [ECPA]:[BzMA]:[$CuCl_2$]:[PMDETA]:[$Sn(EH)_2$] = 1:100:0.2:0.2:0.08 in (1:1.25 solvent to monomer ratio) at 70 °C.

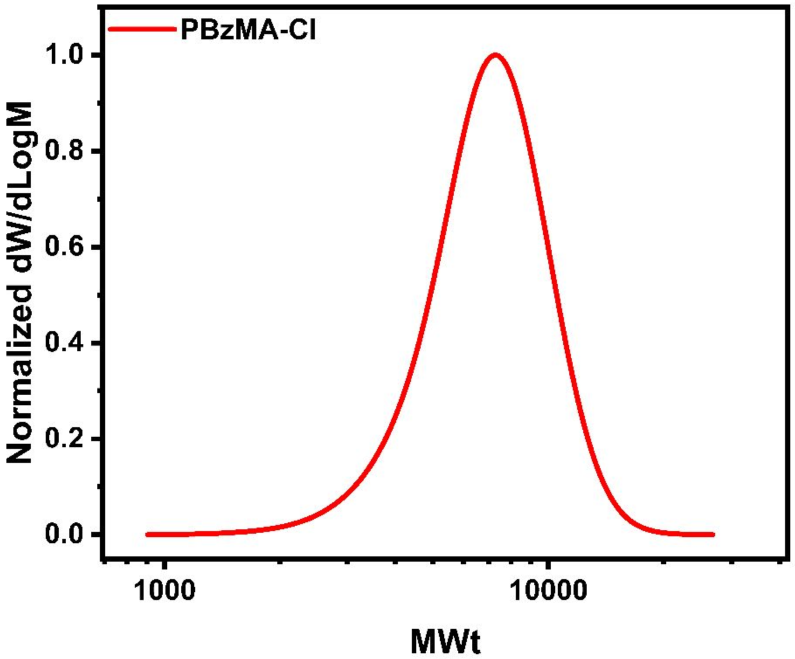

Figure S2: SEC trace of purified PBzMA-Cl. It was synthesized by ARGET-ATRP under the following conditions: [ECPA]:[BzMA]:[$CuCl_2$]:[PMDETA]:[$Sn(EH)_2$] = 1:100:0.2:0.2:0.08 in (1:1.25 solvent to monomer ratio) at 70 °C. The molecular weight was 6400 and the dispersity was 1.15.

Livingness quantification

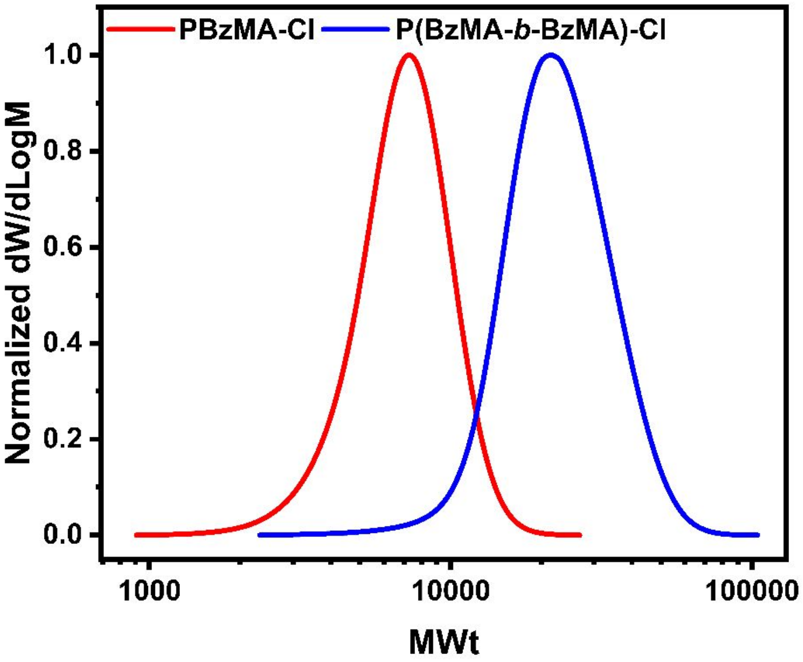

Figure S3: SEC traces of the PBzMA-Cl before and after chain extension with BzMA. Reaction was performed under the following conditions: [PBzMA-Cl]:[BzMA]:[$CuCl_2$]:[PMDETA]:[$Sn(EH)_2$] = 1:200:0.05:0.15:0.20 in acetonitrile (1:1.25 monomer to solvent ratio) at 70 °C. The final molecular weight was 20600 and the dispersity was 1.17.

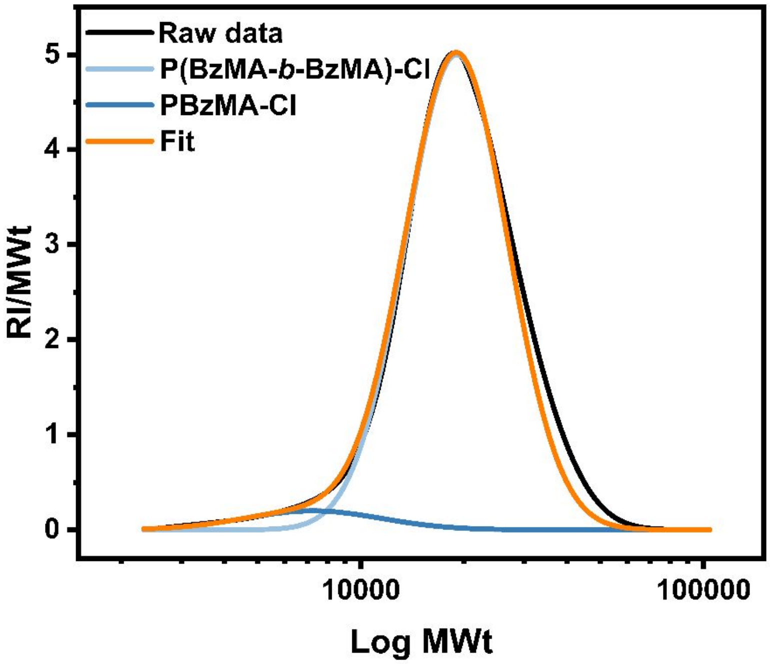

Figure S4: Deconvolution of SEC trace obtained on chain extension of PBzMA-Cl. The livingness was calculated to be 95%, by comparing the integration area of the extended P(BzMA-*b*-BzMA)-Cl versus the unreacted homopolymer (both weight-adjusted). This calculation is based on the methodology reported by Wooley et al.[2]

Deoxygenated depolymerization of PBzMA-Cl

Table S1: The effect of co-solvent on the depolymerization of PBzMA-Cl under deoxygenated conditions.

Entry	Co-solvent (w.r.t. TCB)	Time (min)	Depol. (%) $_{(NMR)}$	Depol. Efficiency (%)
1	0.2% v/v DMF	5	88	93
2		15	89	94
3		30	89	94
4	10% v/v DMF	5	82	86
5		15	83	87
6		30	82	86
7	10% v/v DMSO	5	60	63
8		15	67	71
9		30	70	74

Reaction mixtures were bubbled with nitrogen for 30 minutes prior to depolymerization. Depolymerization efficiency was calculated based on 95% polymer livingness. Reaction conditions were as follows: P-Cl:$CuCl_2$:TPMA = 1:0.22:1.3, [Repeat Unit (RU)] = 50 mM, V_{tot} = 1 mL, solvent = TCB, temperature = 170 °C.

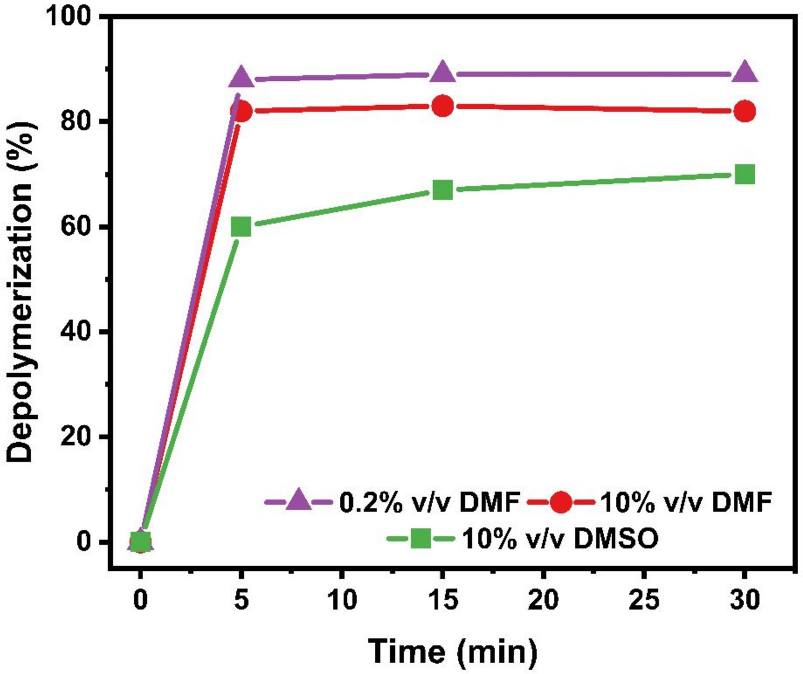

Figure S5: Depolymerization of PBzMA-Cl with various co-solvent contents. Reactions were performed under deoxygenated conditions and conversions were obtained by ^{1}H NMR. Reaction conditions: P-Cl:$CuCl_2$:TPMA = 1:0.22:1.3, [RU] = 50 mM, V_{tot} = 1 mL, solvent = TCB, temperature = 170 °C.

Open-air depolymerization of PBzMA-Cl under previously optimized conditions

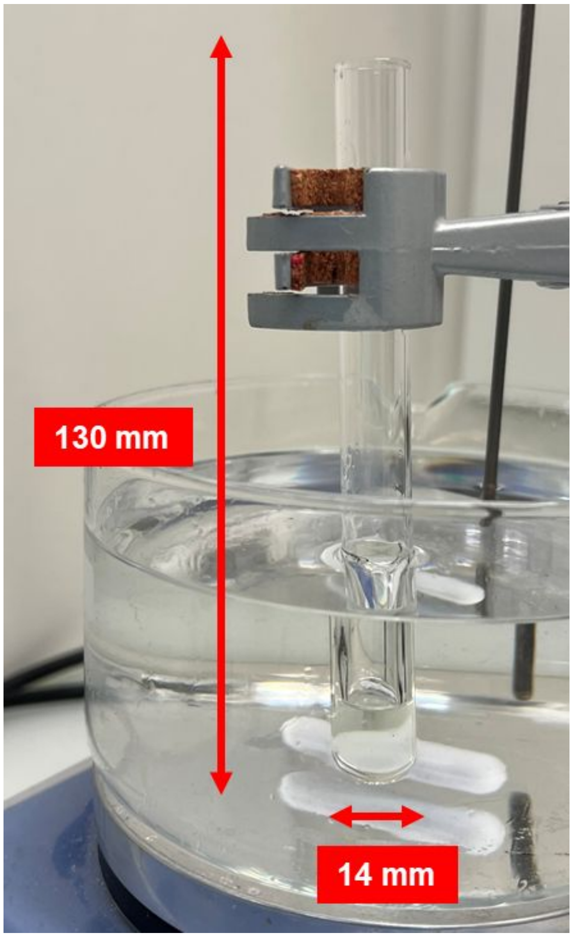

Figure S6: Digital image of experimental set-up for the open-air depolymerization.

Table S2: Assessing the compatibility of previously optimized conditions with the open-air depolymerization of PBzMA-Cl.

Entry	Co-solvent (w.r.t. TCB)	Time (min)	Depol. (%) (NMR)	Depol. Efficiency (%)
1		5	0	0
2	0.2% v/v DMF	15	0	0
3		30	1	1

Extent of open-air depolymerization at various timepoints. Depolymerization efficiency was calculated based on 95% polymer livingness. Reaction conditions were as follows: P-Cl:$CuCl_2$:TPMA = 1:0.22:1.3, [RU] = 50 mM, V_{tot} = 1 mL, solvent = TCB, temperature = 170 °C.

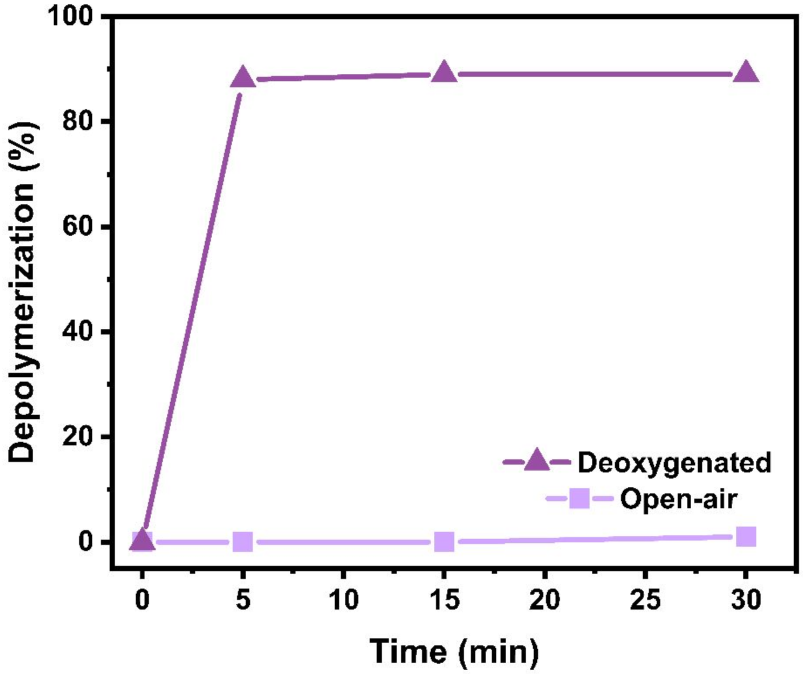

Figure S7: Depolymerization of PBzMA-Cl with DMF as a co-solvent (0.2% v/v). Reactions were performed under either deoxygenated or open-air conditions (P-Cl:$CuCl_2$:TPMA = 1:0.22:1.3, [RU] = 50 mM, V_{tot} = 1 mL, solvent = TCB, temperature = 170 °C).

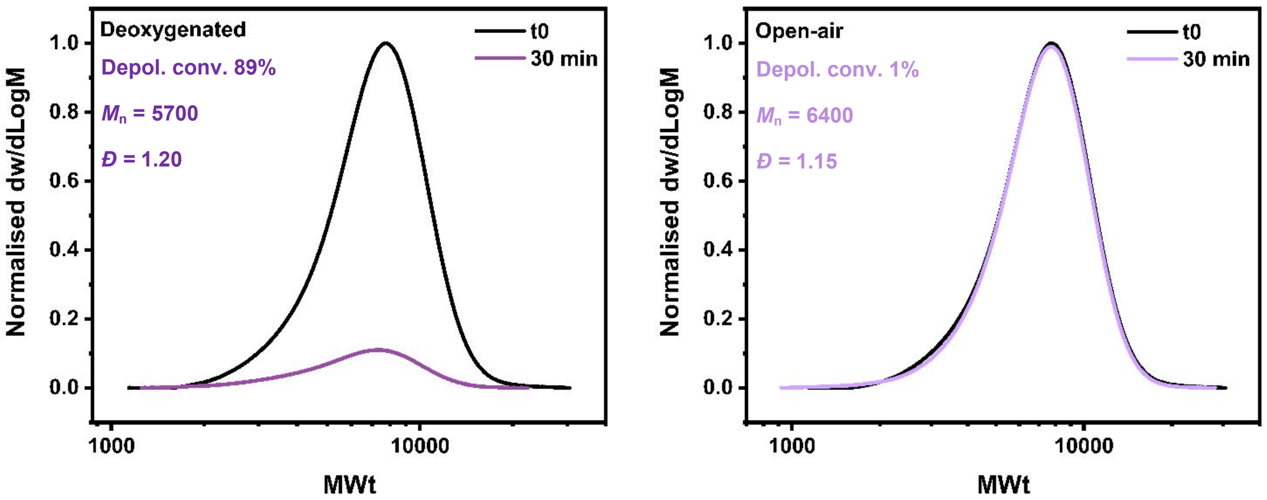

Figure S8: SEC traces of the depolymerization of PBzMA-Cl with DMF as a co-solvent (0.2% v/v). Reactions were performed under either deoxygenated or open-air conditions (P-Cl:$CuCl_2$:TPMA = 1:0.22:1.3, [RU] = 50 mM, V_{tot} = 1 mL, solvent = TCB, temperature = 170 °C). SEC traces are normalized by area based on the depolymerization conversion obtained by ^{1}H NMR.

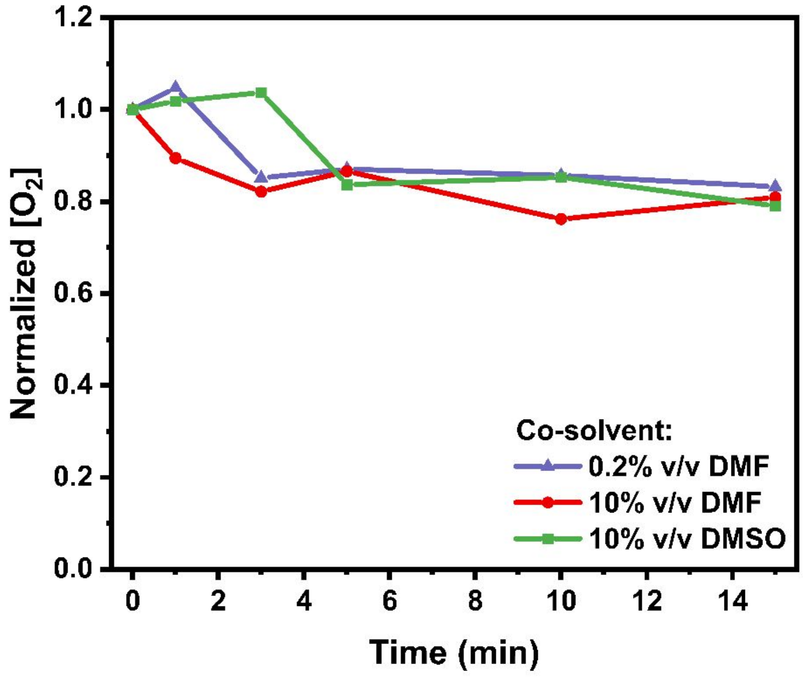

Figure S9: Effect of a high boiling point co-solvent on the oxygen content of reaction over 15 minutes. These experiments were performed in an open vessel and data were collected using an oxygen probe (P-Cl:$CuCl_2$:TPMA = 0:0.22:1.3, V_{tot} = 1 mL, solvent = TCB, temperature = 170 °C).

Open-air depolymerization in the presence of low and high boiling-point co-solvents

Table S3: A comparison of the effect of high and low boiling point cosolvents on the open-air depolymerization of PBzMA-Cl.

Type of co-solvent	Entry	Co-solvent	Boiling point (°C)	Time (min)	Depol. (%) (NMR)	Depol. (%) efficiency	M_n (SEC)	$Đ$ (SEC)
	0	-	-	0	0	0	6400	1.15
High boiling point	1	DMSO	189	5	2	2	-	-
	2			10	2	2	-	-
	3			30	3	3	6400	1.16
	4	DMF	153	5	1	1	-	-
	5			15	5	5	-	-
	6			30	6	6	6300	1.16
Low boiling point	7	MeCN	82	5	62	65	-	-
	8			15	71	75	-	-
	9			30	72	76	6200	1.17
	10	Acetone	56	5	55	58	-	-
	11			15	76	80	-	-
	12			30	76	80	6000	1.18

Extent of open-air depolymerization at various timepoints. Depolymerization efficiency was calculated based on 95% polymer livingness. Reaction conditions were as follows: P-Cl:$CuCl_2$:TPMA = 1:0.22:1.3, [RU] = 50 mM, V_{tot} = 1 mL, co-solvent content = 10% v/v w.r.t TCB, temperature = 170 °C.

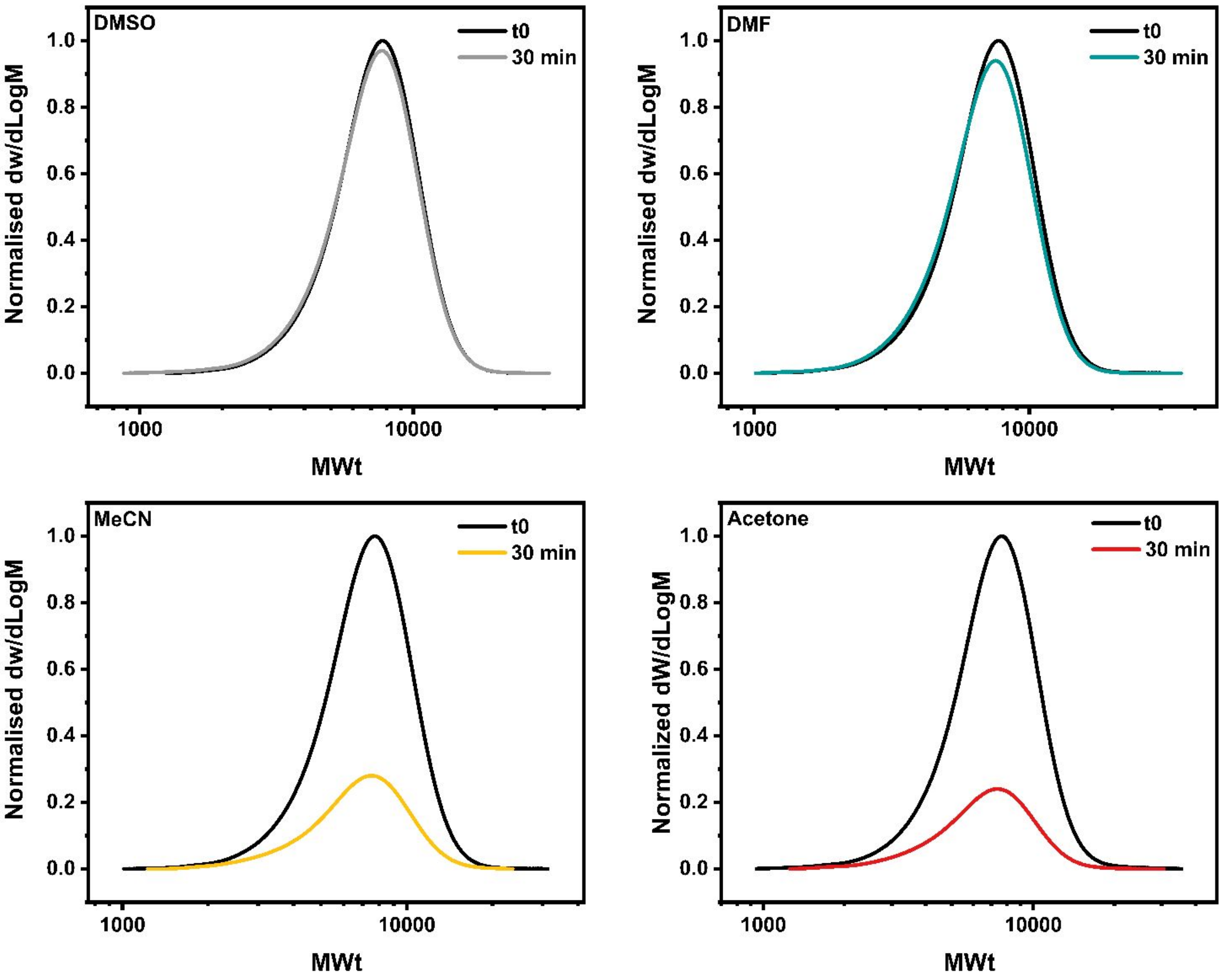

Figure S10: SEC traces of the open-air depolymerization of PBzMA-Cl. Reactions were performed with various co-solvents (P-Cl:$CuCl_2$:TPMA = 1:0.22:1.3, [RU] = 50 mM, V_{tot} = 1 mL, co-solvent content = 10% v/v w.r.t TCB, temperature = 170 °C). SEC traces are normalized by area based on the depolymerization conversion obtained by ^1H NMR.

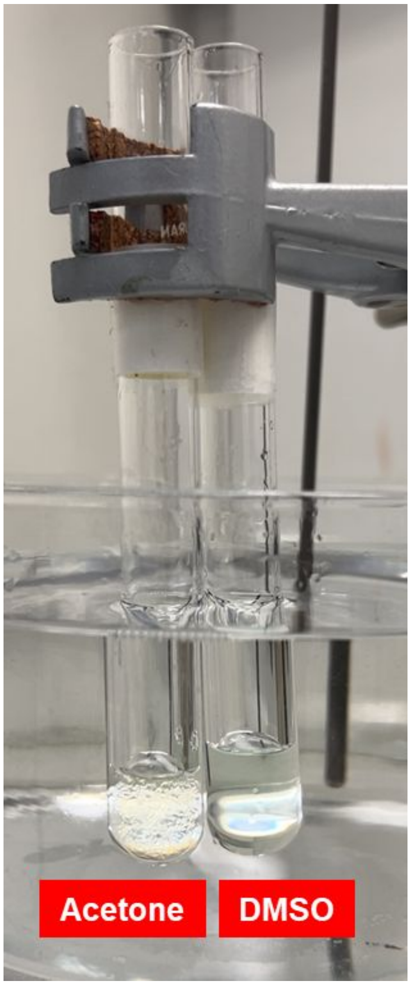

Figure S11: Digital photo comparing the depolymerizations performed with 10% acetone and DMSO. The boiling is observed only in acetone. The remaining 90% of solvent was TCB.

Table S4: A further comparison of the effect of high and low boiling point co-solvents on the open-air depolymerization of PBzMA-Cl.

Type of co-solvent	Entry	Co-solvent	Boiling point (°C)	Time (min)	Depol. (%) (NMR)	Depol. (%) efficiency	$M_{n\ (SEC)}$	$Đ_{(SEC)}$
	0	-	-	0	0	0	6400	1.15
**Low boiling point**	1	THF	66	5	50	53	-	-
	2			15	70	74	-	-
	3			30	69	73	5900	1.19
	4	IPA	82	5	23	24	-	-
	5			15	72	76	-	-
	6			30	71	75	6200	1.19
	7	BuOH	118	5	6	6	-	-
	8			15	67	71	-	-
	9			30	67	71	6300	1.14
**High boiling point**	10	PhCl	132	5	0	0	-	-
	11			15	0	0	-	-
	12			30	1	1	6400	1.16
	13	Xylene	139	5	0	0	-	-
	14			15	1	1	-	-
	15			30	1	1	6400	1.16
	16	TEGDME	275	5	6	6	-	-
	17			15	7	7	-	-
	18			30	7	7	6400	1.16

Extent of open-air depolymerization at various timepoints. Depolymerization efficiency was calculated based on 95% polymer livingness. For xylene and chlorobenzene, 0.2% v/v DMF was used to dissolve the catalyst. Reaction conditions were as follows: P-Cl:$CuCl_2$:TPMA = 1:0.22:1.3, [RU] = 50 mM, V_{tot} = 1 mL, co-solvent content = 10% v/v w.r.t TCB, temperature = 170 °C.

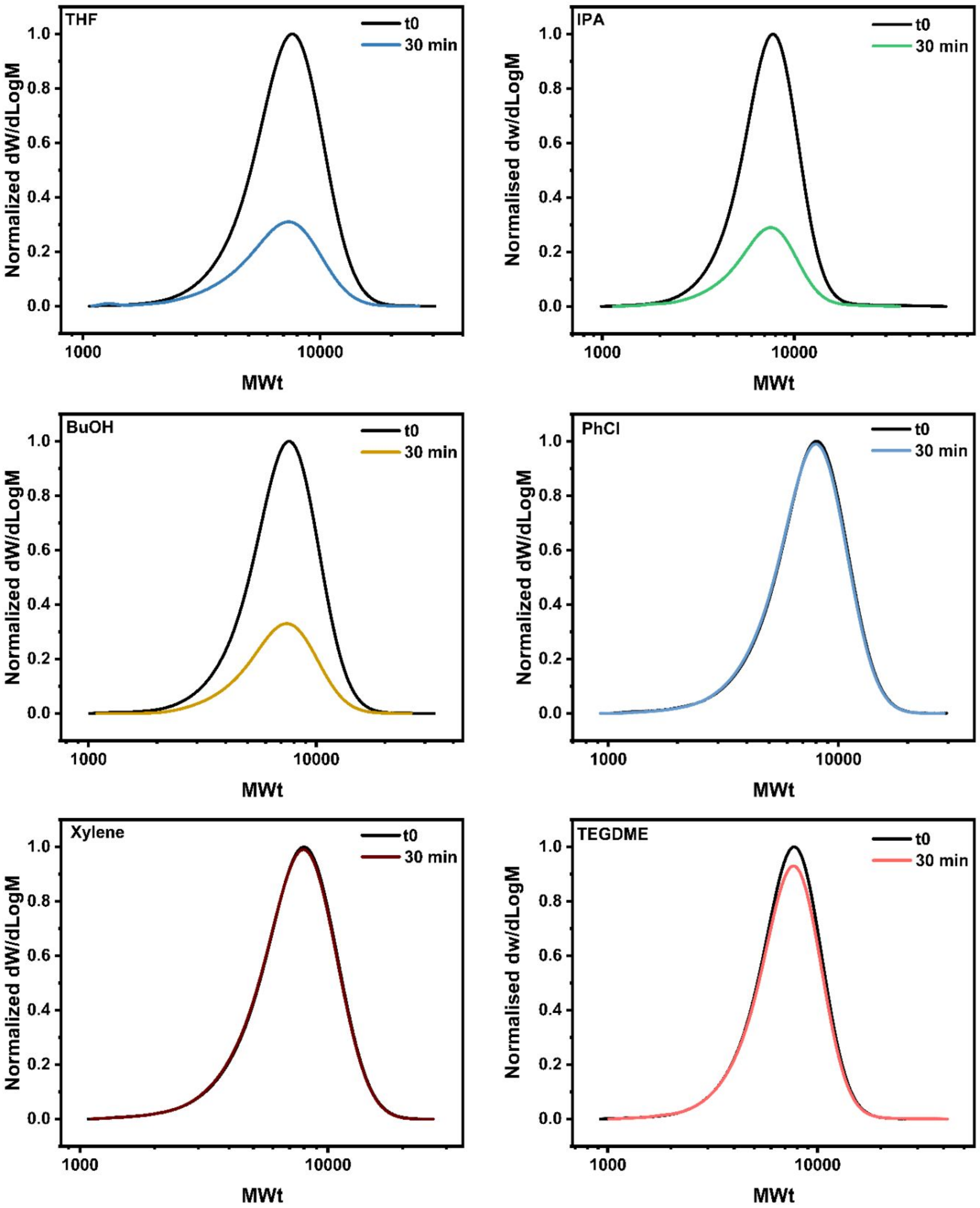

Figure S12: SEC traces of the open-air depolymerization of PBzMA-Cl. Reactions were performed with various co-solvents (P-Cl:$CuCl_2$:TPMA = 1:0.22:1.3, [RU] = 50 mM, V_{tot} = 1 mL, co-solvent content = 10% v/v w.r.t TCB, temperature = 170 °C). SEC traces are normalized by area based on the depolymerization conversion obtained by ^{1}H NMR.

Table S5: The effect of acetone content on the open-air depolymerization of PBzMA-Cl.

Entry	Acetone Content (w.r.t. TCB)	Time (min)	Depol. (%) $_{(NMR)}$	Depol. Efficiency (%)
1		5	5	5
2	5% v/v	15	29	31
3		30	29	31
4		5	55	58
5	10% v/v	15	76	80
6		30	76	80
7		5	79	83
8	20% v/v	15	81	85
9		2	81	85
10		5	87	92
11	30% v/v	15	86	91
12		30	86	91

Extent of open-air depolymerization at various timepoints. Depolymerization efficiency was calculated based on 95% polymer livingness. Reaction conditions were as follows: P-Cl:$CuCl_2$:TPMA = 1:0.22:1.3, [RU] = 50 mM, V_{tot} = 1 mL, solvent = TCB, temperature = 170 °C.

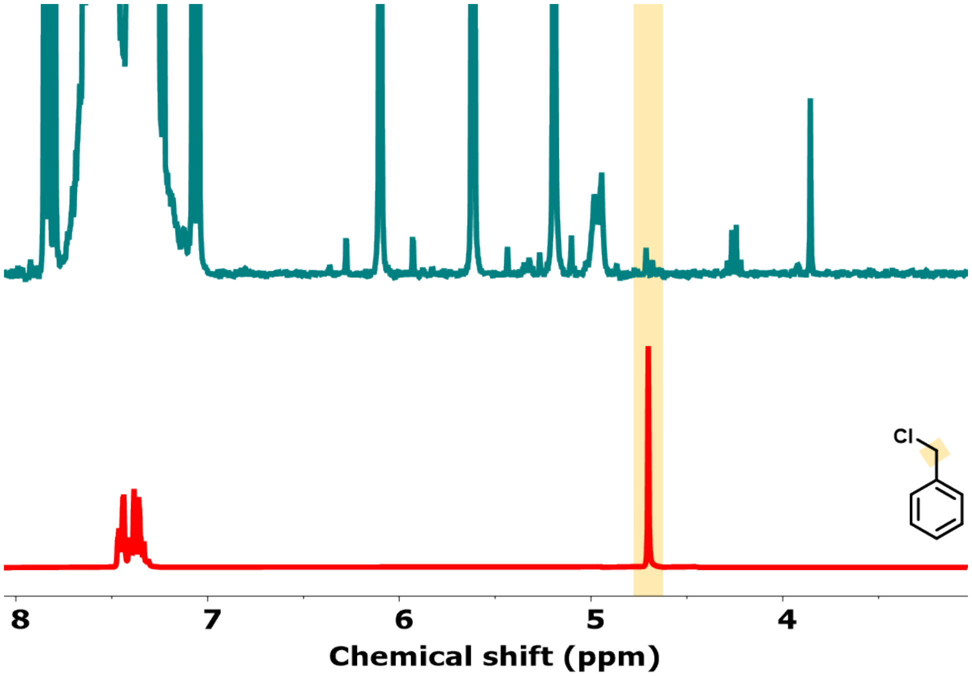

Figure S13: ^{1}H NMR measurements demonstrating that after extended reaction times (30 min), a small amount of lactonization (<5%) had occurred as evidenced by the formation of benzyl chloride.

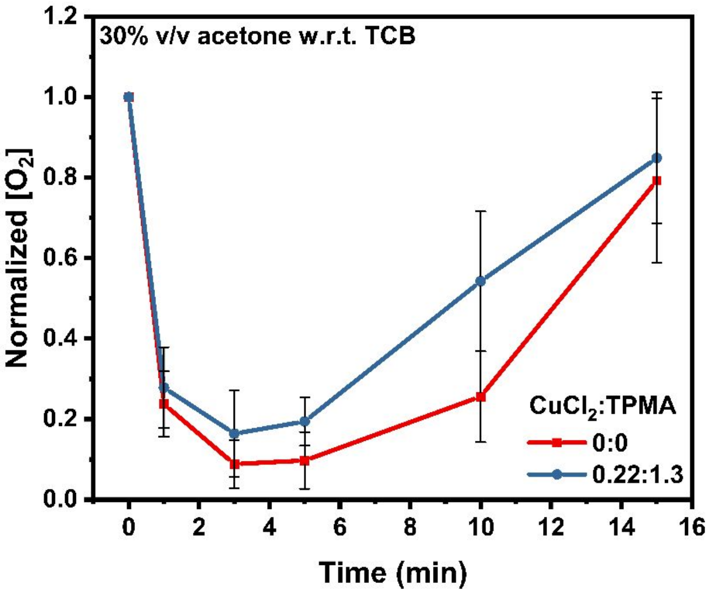

Figure S14: Oxygen probe measurements investigating the effect of the catalyst on the oxygen content reduction over the first 15 minutes of reaction. These experiments were performed in triplicate in an open vessel with 30% v/v acetone w.r.t. TCB in the presence and absence of catalyst.

Depolymerization kinetics of PBzMA-Cl

Table S6: Detailed kinetics for the open-air depolymerization of PBzMA-Cl.

Entry	Time (min)	Depol. (%) (NMR)	Depol. (%) efficiency	M_n (SEC)	$Đ$ (SEC)
1	0	0	0	6400	1.15
2	0.5	1	1	-	-
3	1	2	2	-	-
4	1.5	2	2	6200	1.17
5	2	3	3	-	-
6	3	44	46	5200	1.23
7	5	84	88	-	-
8	10	85	89	-	-
9	15	85	89	5300	1.24

Extent of open-air depolymerization at various timepoints. Depolymerization efficiency was calculated based on 95% polymer livingness. Reaction conditions were as follows: P-Cl:$CuCl_2$:TPMA = 1:0.22:1.3, [RU] = 50 mM, V_{tot} = 1 mL, acetone content = 30% v/v w.r.t TCB, temperature = 170 °C.

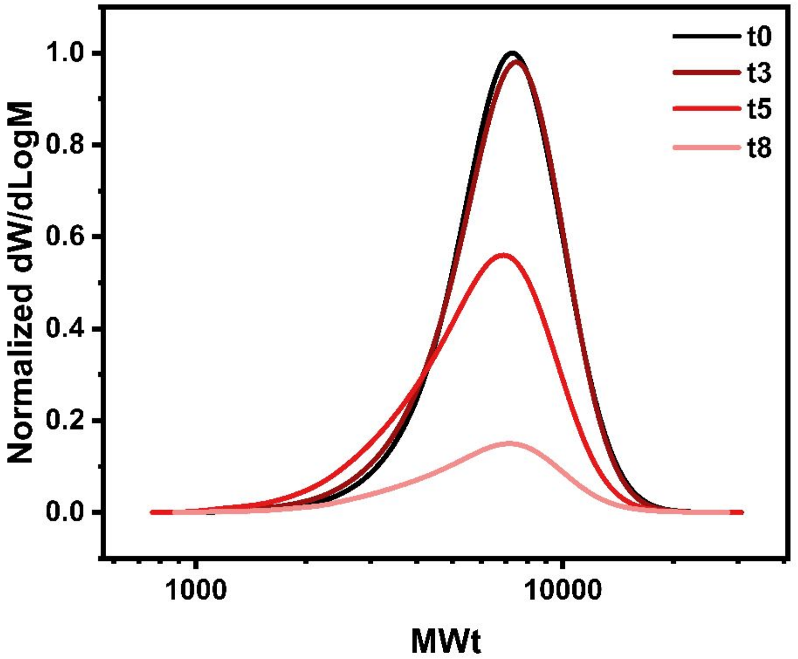

Figure S15: Selected SEC traces for the open-air depolymerization of PBzMA-Cl. Reaction was performed under the optimized conditions of P-Cl:$CuCl_2$:TPMA = 1:0.22:1.3 with a repeat unit concentration of 50 mM, V_{tot} of 1 mL, acetone content of 30% v/v w.r.t TCB and a temperature of 170 °C. SEC traces are normalized by area based on the depolymerization conversion obtained by ^{1}H NMR.

Expanding the polymer scope of open-air depolymerization

Polymer synthesis of higher molecular weight PBzMA-Cl by ARGET-ATRP

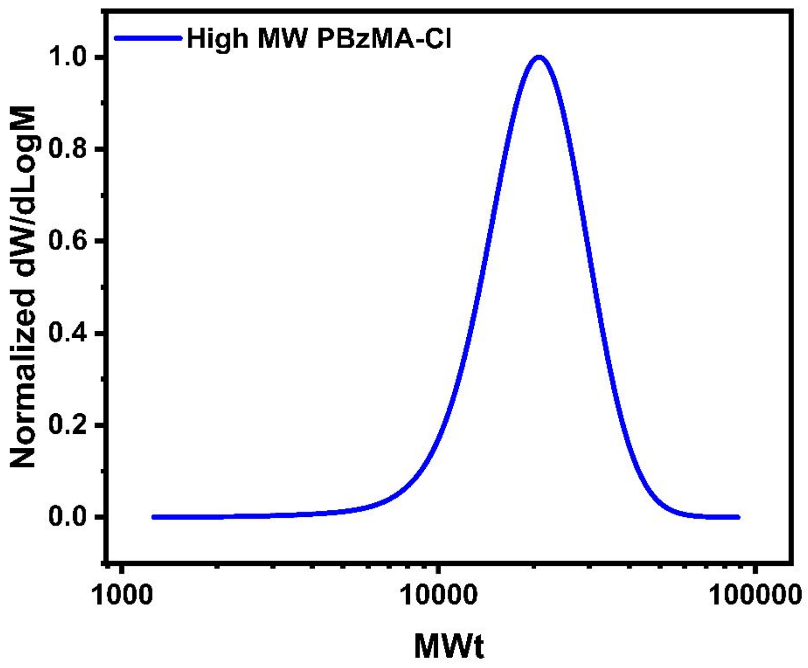

Figure S16: SEC trace of purified PBzMA-Cl. It was synthesized by ARGET-ATRP under the following conditions: [ECPA]:[BzMA]:[$CuCl_2$]:[PMDETA]:[Sn(EH)$_2$] = 1:240:0.1:0.15:0.20 in (0.5:1 solvent to monomer ratio) at 70 °C. The molecular weight was 18000 and the dispersity was 1.17.

Depolymerization of higher molecular weight PBzMA-Cl

Table S7: Experimental data obtained for the open-air depolymerization of higher molecular weight poly(benzyl methacylate).

Entry	PBzMA-Cl:$CuCl_2$:TPMA	Time (min)	Depol. (%) (NMR)
1		5	27
2	1:0.22:1.3	15	62
3		30	57
4		5	15
5	1:0.66:3.9	15	68
6		30	70
7		5	76
8	1:1.2:7	15	72
9		30	71

Extent of open-air depolymerization at various timepoints. Reaction conditions were as follows: [RU] = 50 mM, V_{tot} = 1 mL, 30% v/v acetone w.r.t. TCB, temperature = 170 °C.

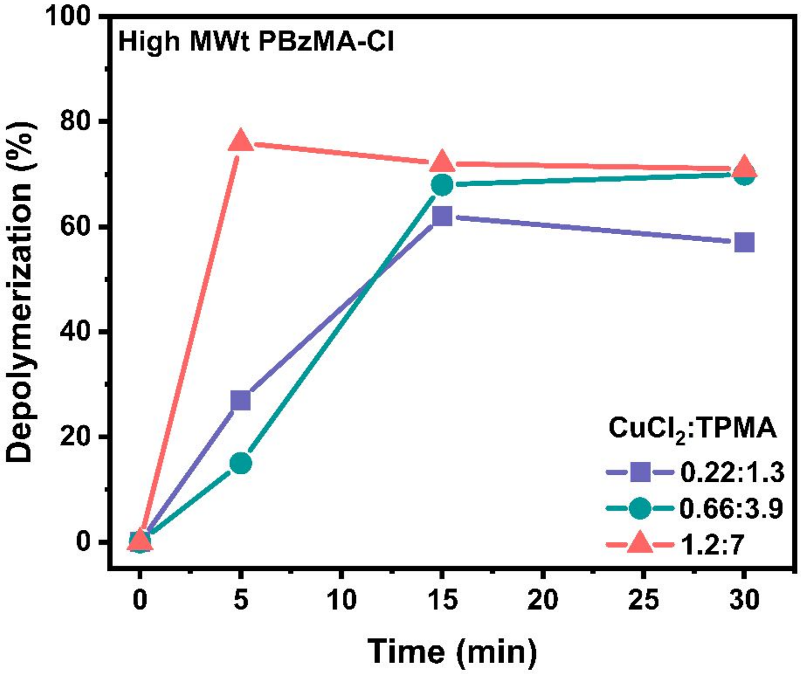

Figure S17: Depolymerization extent of higher molecular weight PBzMA-Cl at various timepoints, as measured by ^{1}H NMR. All reactions were performed in an open vessel with acetone as a co-solvent ([RU] = 50 mM, V_{tot} = 1 mL, acetone content = 30% v/v w.r.t TCB, temperature = 170 °C).

Polymer synthesis of PMMA-Cl by ARGET-ATRP

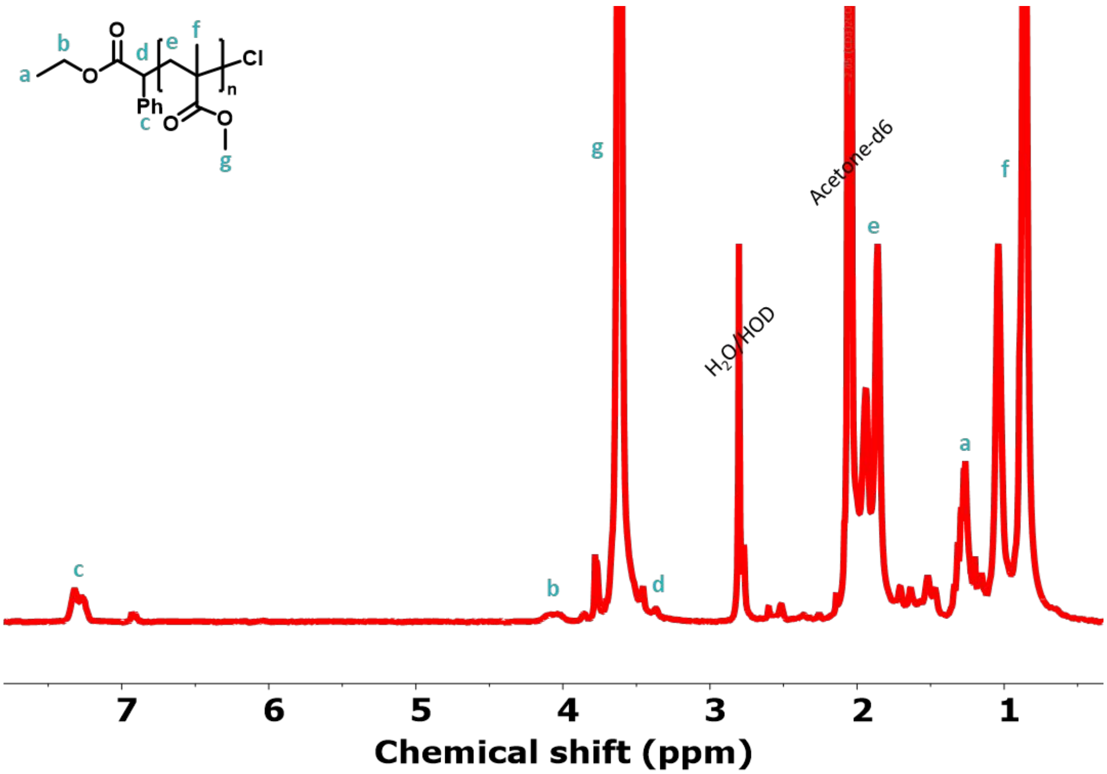

Figure S18: ^{1}H NMR spectra of PMMA-Cl after purification. It was synthesized by ARGET-ATRP under the following conditions: [ECPA]:[MMA]:[$CuCl_2$]:[PMDETA]:[$Sn(EH)_2$] = 1:100:0.1:0.15:0.20 in (0.5:1 solvent to monomer ratio) at 70 °C.

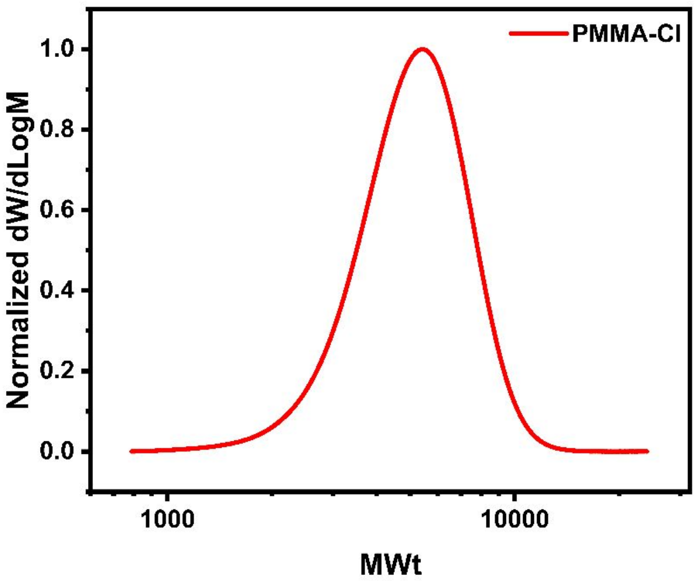

Figure S19: SEC trace of purified PMMA-Cl. It was synthesized by ARGET-ATRP under the following conditions: [ECPA]:[MMA]:[$CuCl_2$]:[PMDETA]:[$Sn(EH)_2$] = 1:100:0.1:0.15:0.20 in (0.5:1 solvent to monomer ratio) at 70 °C. The molecular weight was 4700 and the dispersity was 1.15.

Polymer synthesis of PBMA-Cl by ARGET-ATRP

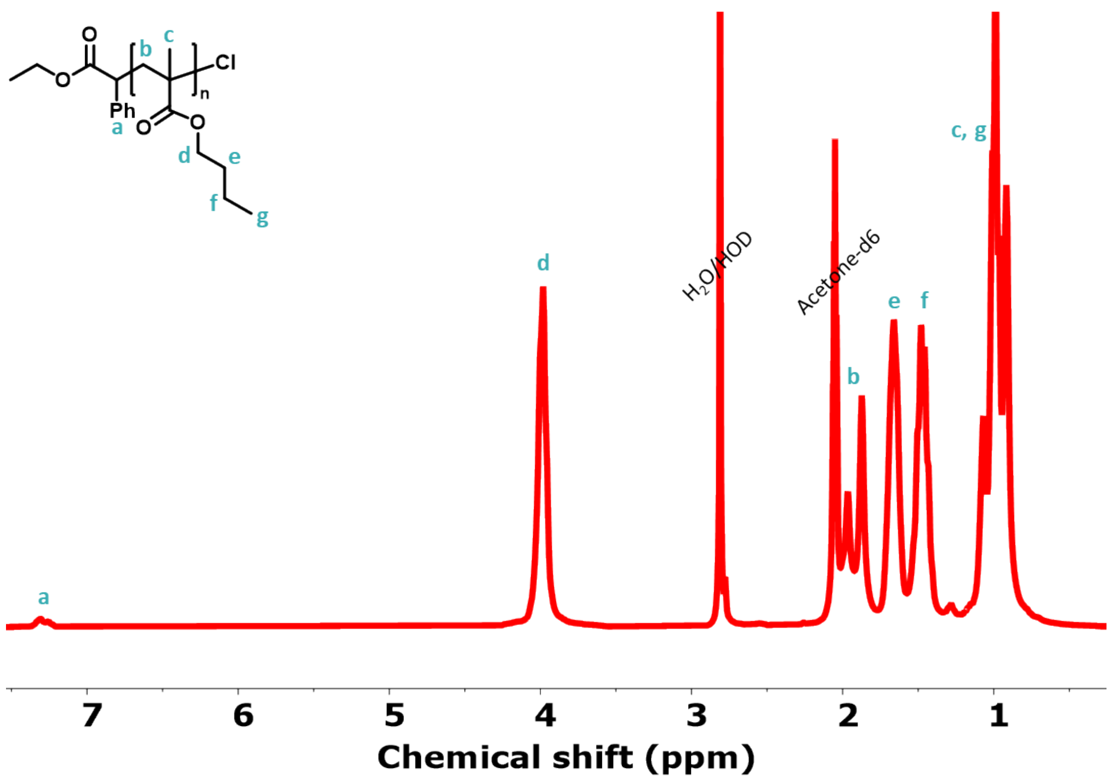

Figure S20: ^{1}H NMR spectra of PBMA-Cl after purification. It was synthesized by ARGET-ATRP under the following conditions: [ECPA]:[BMA]:[$CuCl_2$]:[PMDETA]:[$Sn(EH)_2$] = 1:100:0.1:0.15:0.20 in (0.5:1 solvent to monomer ratio) at 70 °C.

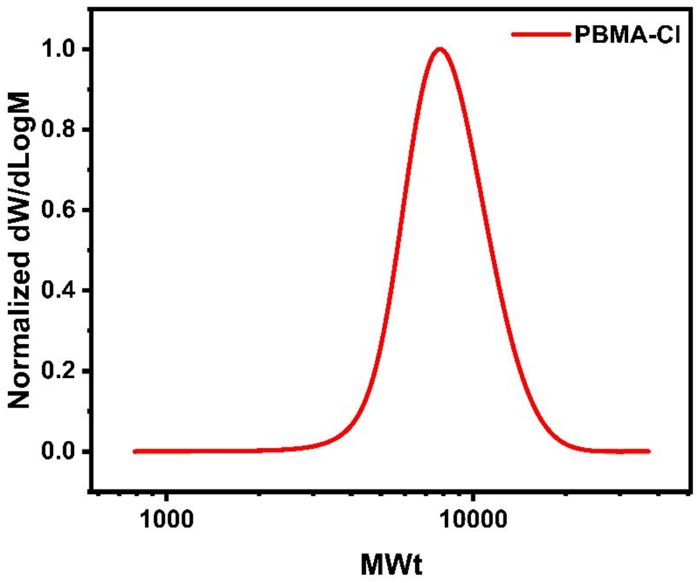

Figure S21: SEC trace of purified PBMA-Cl. It was synthesized by ARGET-ATRP under the following conditions: [ECPA]:[BMA]:[$CuCl_2$]:[PMDETA]:[$Sn(EH)_2$] = 1:100:0.1:0.15:0.20 in (0.5:1 solvent to monomer ratio) at 70 °C. The molecular weight was 7700 and the dispersity was 1.11.

Depolymerization of PMMA-Cl and PBMA-Cl

Table S8: Experimental data obtained for the open-air depolymerization of various alternative polymethacrylates.

Entry	Polymer	Time (min)	Depol. (%) $_{(NMR)}$
1	PMMA-Cl	5	87
2		15	89
3		30	88
4	PBMA-Cl	5	77
5		15	88
6		30	86

Extent of open-air depolymerization at various timepoints. Reaction conditions were as follows: P-Cl:$CuCl_2$:TPMA = 1:0.22:1.3, [RU] = 50 mM, V_{tot} = 1 mL, 30% v/v acetone w.r.t. TCB, temperature = 170 °C.

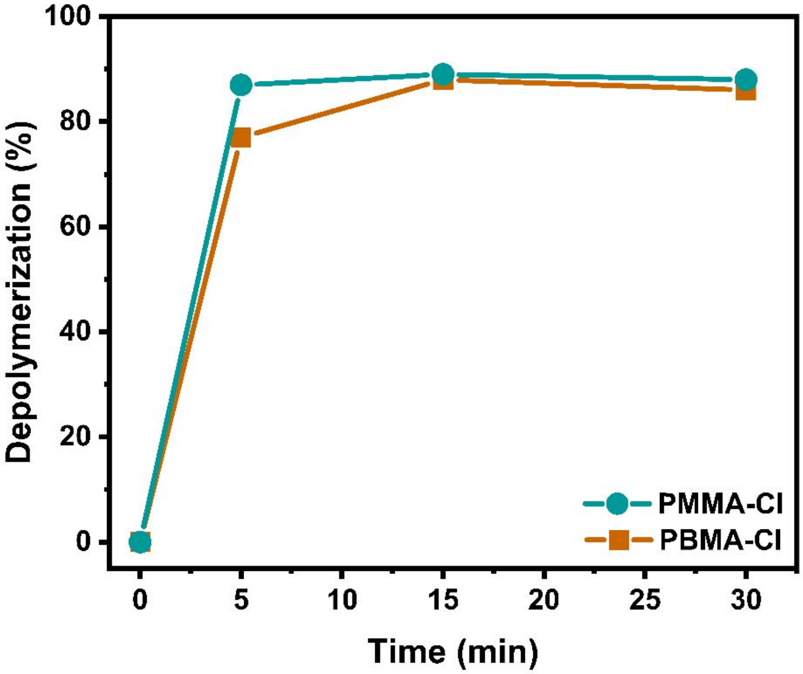

Figure S22: Depolymerization extent of PMMA-Cl and PBMA-Cl at various timepoints, as measured by ^{1}H NMR. All reactions were performed in an open vessel with acetone as a co-solvent (P-Cl:$CuCl_2$:L = 1:0.22:1.3, [RU] = 50 mM, V_{tot} = 1 mL, acetone content = 30% v/v w.r.t TCB, temperature = 170 °C).

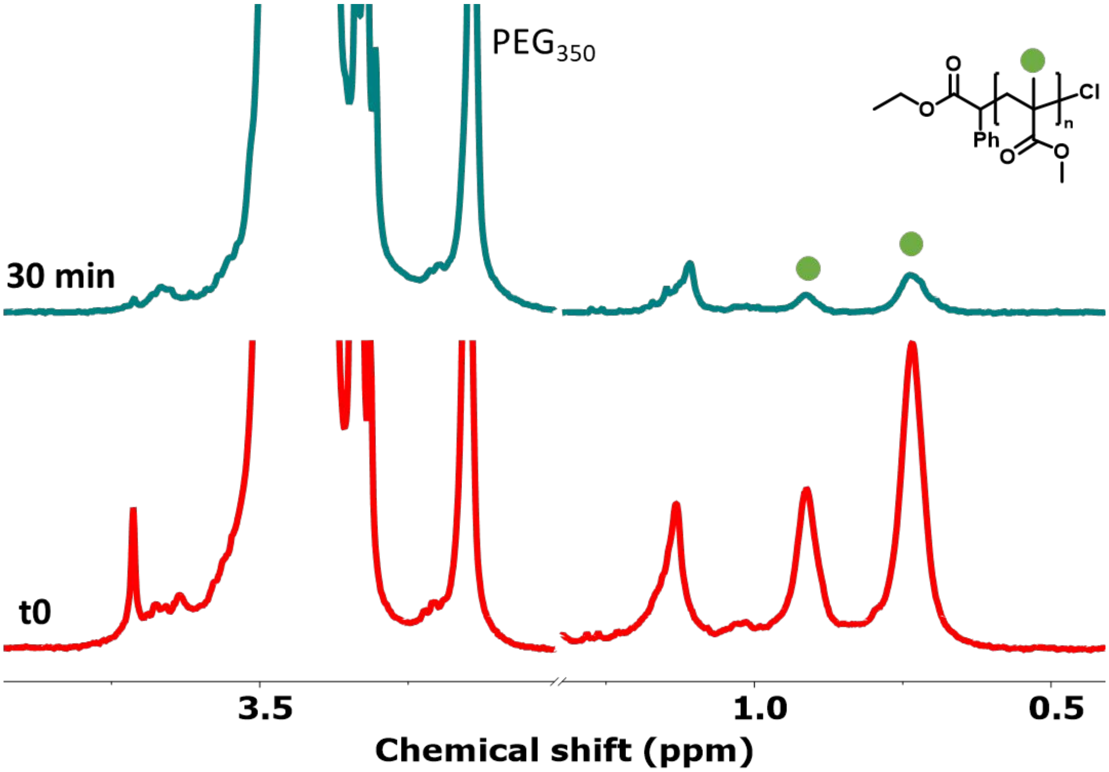

Figure S23: ^{1}H NMR spectra of PMMA-Cl before (bottom) and after (top) depolymerization. The depolymerization conversion was 88%.

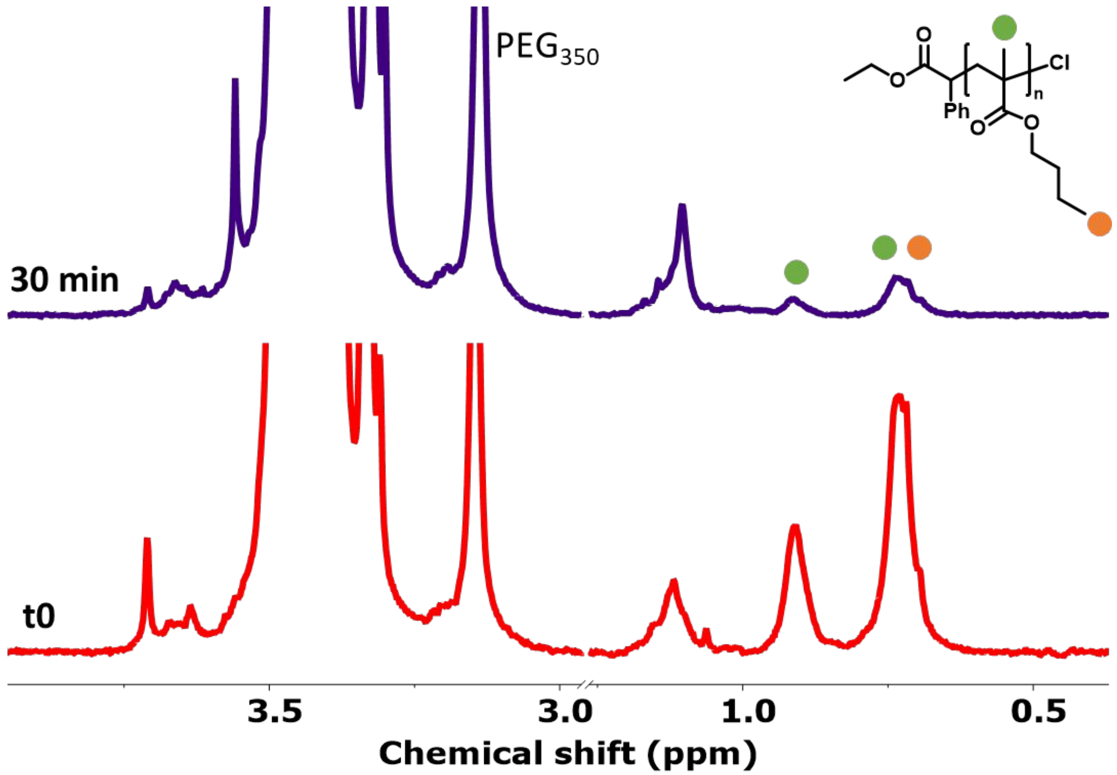

Figure S24: ^{1}H NMR spectra of PBMA-Cl before (bottom) and after (top) depolymerization. The depolymerization conversion was 86%.

Expanding the ligand compatibility of open-air depolymerization

Table S9: The effect of ligand choice on the open-air depolymerization of PBzMA-Cl.

Entry	Ligand	Time (min)	Depol. (%) (NMR)	Depol. (%) efficiency
1		5	7	7
2	dNbpy	15	55	58
3		30	52	55
4		5	60	63
5	HMTETA	15	70	74
6		30	71	75
7		5	65	68
8	PMDETA	15	76	80
9		30	75	79
10		5	87	92
11	TPMA	15	86	91
12		30	86	91
13		5	78	82
14	Me_6TREN	15	81	85
15		30	81	85

Extent of open-air depolymerization at various timepoints. Depolymerization efficiency was calculated based on 95% polymer livingness. Reaction conditions were as follows: P-Cl:$CuCl_2$:L = 1:0.22:1.3, [RU] = 50 mM, V_{tot} = 1 mL, acetone content = 30% v/v w.r.t TCB, temperature = 170 °C.

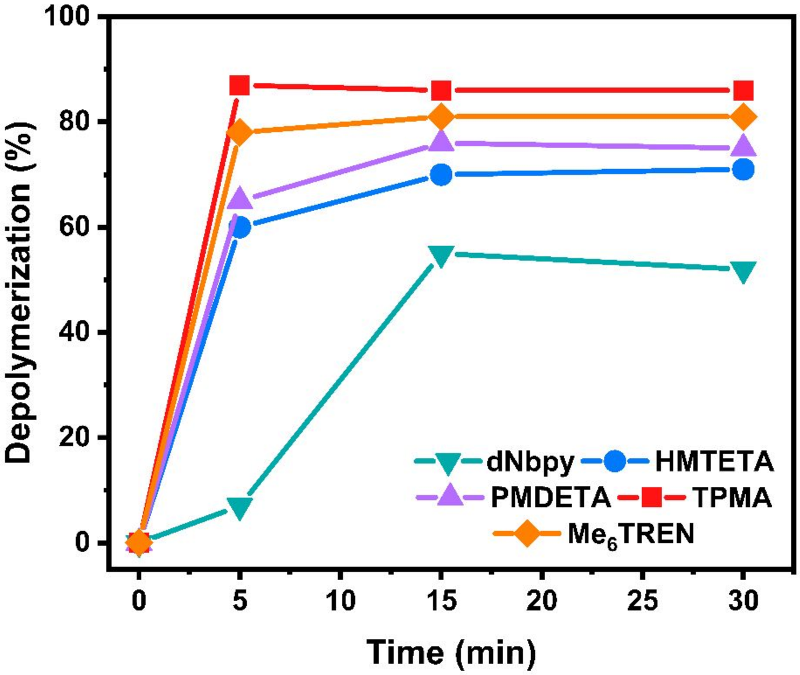

Figure S25: Depolymerization extent of PBzMA-Cl when various ligands were employed, as measured by ^1H NMR. All reactions were performed in an open vessel with acetone as a co-solvent (P-Cl:$CuCl_2$:L = 1:0.22:1.3, [RU] = 50 mM, V_{tot} = 1 mL, acetone content = 30% v/v w.r.t TCB, temperature = 170 °C).

The effect of the repeat unit concentration on the open-air depolymerization

Table S10: The effect of repeat unit concentration on the open-air depolymerization of PBzMA-Cl.

Entry	[RU] (mM)	Time (min)	Depol. (%) $_{(NMR)}$	Depol. (%) efficiency
1		5	87	92
2	50	15	86	91
3		30	86	91
4		5	86	91
5	250	15	81	85
6		30	81	85
7		5	81	85
8	500	15	79	83
9		30	79	83
10		5	76	80
11	750	15	76	80
12		30	75	79
13		5	73	77
14	1000	15	72	76
15		30	72	76

Extent of open-air depolymerization at various timepoints. Depolymerization efficiency was calculated based on 95% polymer livingness. Reaction conditions were as follows: P-Cl:$CuCl_2$:TPMA = 1:0.22:1.3, V_{tot} = 1 mL, acetone content = 30% v/v w.r.t TCB, temperature = 170 °C.

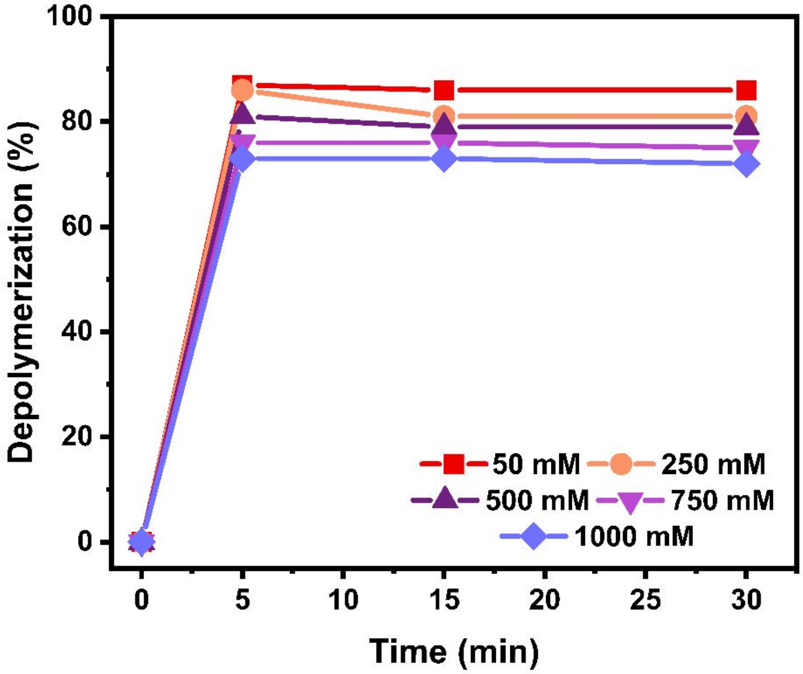

Figure S26: ^{1}H NMR data illustrating the effect of repeat unit concentration on the depolymerization conversion with acetone as a co-solvent under open-air conditions (P-Cl:$CuCl_2$:TPMA = 1:0.22:1.3, [RU] = 50 mM, V_{tot} = 1 mL, acetone content = 30% v/v w.r.t TCB, temperature = 170 °C).

References

(1) Ciampolini, M.; Nardi, N. Five-Coordinated High-Spin Complexes of Bivalent Cobalt, Nickel, andCopper with Tris (2-dimethylaminoethyl) amine. *Inorg. Chem.* **1966**, *5*, 1, 41-44

(2) Bartels, J. W.; Cauët, S. I.; Billings, P. L.; Lin, L. Y.; Zhu, J.; Fidge, C.; Pochan, D. J.; Wooley, K. L. Evaluation of Isoprene Chain Extension from PEO Macromolecular Chain Transfer Agents for the Preparation of Dual, Invertible Block Copolymer Nanoassemblies. *Macromolecules* **2010**, *43*, 17, 7128-7138
